# ALKBH5 promotes lung fibroblast activation and silica-induced pulmonary fibrosis through miR-320a-3p and FOXM1

**DOI:** 10.1186/s11658-022-00329-5

**Published:** 2022-03-12

**Authors:** Wenqing Sun, Yan Li, Dongyu Ma, Yi Liu, Qi Xu, Demin Cheng, Guanru Li, Chunhui Ni

**Affiliations:** 1 Department of Occupational Medical and Environmental Health, Key Laboratory of Modern Toxicology of Ministry of Education, Center for Global Health, School of Public Health, Nanjing Medical University, Nanjing, 211166 China; 2 Gusu School, Nanjing Medical University, Nanjing, 211166 China

**Keywords:** Silicosis, ALKBH5, miR-320a-3p, FOXM1, m^6^A

## Abstract

**Background:**

*N*^6^-methyladenosine (m^6^A) is the most common and abundant internal modification of RNA. Its critical functions in multiple physiological and pathological processes have been reported. However, the role of m^6^A in silica-induced pulmonary fibrosis has not been fully elucidated. AlkB homolog 5 (ALKBH5), a well-known m^6^A demethylase, is upregulated in the silica-induced mouse pulmonary fibrosis model. Here, we sought to investigate the function of ALKBH5 in pulmonary fibrosis triggered by silica inhalation.

**Methods:**

We performed studies with fibroblast cell lines and silica-induced mouse pulmonary fibrosis models. The expression of ALKBH5, miR-320a-3p, and forkhead box protein M1 (FOXM1) was determined by quantitative real-time polymerase chain reaction (qRT-PCR) analysis. RNA immunoprecipitation (RIP) assays and m^6^A RNA immunoprecipitation assays (MeRIP), western bolt, immunofluorescence assays, and 5-ethynyl-2'-deoxyuridine (EdU) fluorescence staining were performed to explore the roles of ALKBH5, miR-320a-3p, and FOXM1 in fibroblast activation.

**Results:**

ALKBH5 expression was increased in silica-inhaled mouse lung tissues and transforming growth factor (TGF)-β1-stimulated fibroblasts. Moreover, ALKBH5 knockdown exerted antifibrotic effects in vitro. Simultaneously, downregulation of ALKBH5 elevated miR-320a-3p but decreased pri-miR-320a-3p. Mechanically, ALKBH5 demethylated pri-miR-320a-3p, thus blocking the microprocessor protein DGCR8 from interacting with pri-miR-320a-3p and leading to mature process blockage of pri-miR-320a-3p. We further demonstrated that miR-320a-3p could regulate fibrosis by targeting FOXM1 messenger RNA (mRNA) 3′-untranslated region (UTR). Notably, our study also verified that ALKBH5 could also directly regulate FOXM1 in an m^6^A-dependent manner.

**Conclusions:**

Our findings suggest that ALKBH5 promotes silica-induced lung fibrosis via the miR-320a-3p/FOXM1 axis or targeting FOXM1 directly. Approaches aimed at ALKBH5 may be efficacious in treating lung fibrosis.

**Supplementary Information:**

The online version contains supplementary material available at 10.1186/s11658-022-00329-5.

## Background

Silica is one of the most abundant natural minerals on Earth, and exposure to breathable crystalline silica can cause silicosis. Silicosis is characterized by irreversible lung fibrosis [1] and is one of the most widespread occupational diseases in the world, especially in low- and middle-income countries [2]. Unfortunately, silicosis is a latently fatal lung disease and there are no effective treatments because of its sophisticated pathogenesis [3, 4]. In-depth exploration of the mechanisms of silica-induced pulmonary fibrosis could provide a solid theoretical basis for its early diagnosis, intervention, and treatment. In a general scenario, fibroblasts proliferate and differentiate into myofibroblasts in response to cytokines and growth factors, a key event in silicosis that is known as fibroblast activation. Activated fibroblasts display distinct features such as overexpression of alpha-smooth muscle actin (α-SMA) and excessive secretion of extracellular matrix (ECM) proteins [5]. Strong evidence supports that transforming growth factor-β1 (TGF-β1) functions as the primary fibrogenic cytokine in numerous fibrosis diseases [6, 7] via both canonical (Smad-based) and non-canonical (non-Smad-based) signaling pathways. Several of these pathways may offer the potential for pharmacologic intervention [7]. Thus, there is always great demand to investigate potential regulatory molecules and mechanisms that influence fibroblast activation in silica-induced pulmonary fibrosis.

MicroRNAs (miRNAs) are small noncoding RNA molecules, regulating target genes through posttranscriptional inhibition and destabilization [8]. Intensive investigations have highlighted the role of microRNAs in cell differentiation and proliferation, and cell–cell interaction [9, 10]. Our group has reported significant change of some microRNAs in silica-induced pulmonary fibrosis [11], and most of them also showed aberrant function in the idiopathic pulmonary fibrosis process [12]. One of the crucial regulatory mechanisms of miRNA expression is control of miRNA processing [13]. DGCR8 (a member of the RNase III family) recognizes the specific structure of precursor molecules (pri-miRNAs), which is the first step during miRNA biogenesis, and then recruits Drosha ribonuclease III (DROSHA) [14, 15]. A microprocessor complex consisting of DGCR8 and DROSHA cleaves the RNA duplex to yield the pre-miRNA product [15, 16]. Hence, controlling the level of DGCR8 or its recognition of miRNAs is a promising method to regulate the spatiotemporal expression patterns of miRNAs. Recent studies have demonstrated that *N*^6^-methyladenosine (m^6^A) RNA methylation may influence DGCR8-mediated miRNA biogenesis [14, 16]. A previous study reported that a fraction of mature miRNAs, including the miR-320 family, harbors methyl markers [17]. miR-320a-3p, one member of the miR320 family, has been verified to act as a vital regulator in various diseases [18, 19], but its functional role and the underlying mechanism in silica-induced pulmonary fibrosis remain largely unknown.

The effectors in m^6^A pathways include “writers,” “erasers,” and “readers,” which together determine the effects of m^6^A [20]. To date, m^6^A has been considered the most abundant RNA modification in eukaryotic cells [21]. AlkB homolog 5 (ALKBH5) is a well-known m^6^A demethylase (also called m^6^A “eraser”) that is positively expressed in human lung tissue and has aroused significant biological and pharmacological interest [22]. Substantial studies have demonstrated recently that ALKBH5 is involved in biological processes by modulating demethylation of RNA [23–26]. At noncoding RNA levels, knockdown of ALKBH5 could increase m^6^A modification of miRNAs [27]. It is well documented that ALKBH5 affects expression of miR-107 [26] and miR-7 [24], but the concrete mechanisms remain to be elucidated. In our earlier work, miR-320a-3p was downregulated in silica-induced lung fibrosis, leading us to hypothesize that ALKBH5 may modulate miR-320a-3p processing in an m^6^A-dependent manner.

To validate this hypothesize, we demonstrate herein that ALKBH5 is upregulated and plays a profibrotic role in silica-induced lung fibrosis and TGF-β1-stimulated fibroblast activation. We also reveal that ALKBH5 can block the pri-miR-320a-3p process in an m^6^A-dependent manner. Furthermore, we verify the involvement of miR-320a-3p in the regulation of pulmonary fibrosis via targeting FOXM1, the latter of which we have previously validated to promote silica-induced pulmonary fibrosis [28]. Interestingly, we find that ALKBH5 can also target FOXM1 directly in pulmonary fibrosis. These findings may provide promising strategies for pulmonary fibrosis therapy.

## Materials and methods

### Animal models

Male C57BL/6 mice (19–21 g) were purchased from the Animals Core Facility of Nanjing Medical University (Nanjing, China). The detailed operation steps of the silica-induced pulmonary fibrosis model were described previously [11, 29].

For the mouse model of miR-320a-3p overexpression, a total of 24 male C57BL/6 mice were divided randomly into four groups (*n* = 6 in each group): saline, silica, silica plus AAV9-miR-NC, and silica plus AAV9-miR-320a-3p. The mice in the silica plus AAV9-miR-NC/AAV9-miR-320a-3p groups were anesthetized using the same method, then administered intratracheally 50 μl AAV9-miR-NC/AAV9-miR-320a-3p per mouse at a titer of 8 × 10^12^ v. g./ml. Three weeks later, these mice were treated in the same way using anesthesia, saline, and silica as mentioned above. Subsequently, after 4 weeks, the mice were sacrificed, and the lungs were isolated and frozen at −80 ℃ for further study.

All animal experiments were conducted according to the guidelines of the Institutional Animal Care and Use Committee and approved by the Institutional Ethics Committee of Nanjing Medical University (IACUC-2010037).

### Histopathology and tissue hydroxyproline content assay

Hematoxylin and eosin (H&E) staining was performed using standard procedures in cooperation with Servicebio Co., Ltd. (Wuhan, China). The classification criteria were described in detailed previously [29]. Briefly, the fresh right lungs of mice were soaked in 4% paraformaldehyde overnight. Then, the tissues were embedded in paraffin and sectioned into 5-μm-thick slices. The sections were subsequently stained with hematoxylin and eosin and scanned by scanning electron microscopy (Pannoramic). Pathological changes in the lungs were measured according to the degree of alveolar wall thickening, cellular proliferation, inflammatory lesions, collagen deposition, and extent of fibrotic lesions using the following classification criteria: Lesion severity: 0 = nothing/zero, 1 = marginal, 2 = slight, 3 = moderate, 4 = severe, 5 = very severe; lesion distribution: 0 = absent, 1 = rare/occasional (10% of the lung area), 2 = sparse/ limited (10–25% of lung area), 3 = moderate (25–50% of lung area), 4 = extensive/widespread (50–75% of lung area), 5 = very extensive/predominant (over 75% of lung area). The statistical results are presented in Additional file 6: Table S1.

A hydroxyproline assay kit (A030-2, Jincheng Bioengineering Institute, Nanjing, China) was used to detect the degree of collagen deposition following the instructions. Briefly, accurately weighed lung tissues were homogenized in 1 ml hydrolysate for 20 min at 95 °C, and 1 μl indicator as added to the cooling homogenate . Then 1 ml pH A solution was added, and the homogenate turned red. Another pH B solution was added dropwise until the color turned yellow–green, yielding a final pH of 6.0–6.8. The homogenate was then diluted to 10 ml, and 3 ml of the diluent was mixed with 30 mg activated carbon, followed by centrifugation at 3500 rpm for 3 min. Finally, the supernatant was examined by spectrophotometer at 550 nm, and the hydroxyproline content was calculated according to the instructions.

### Cell culture and treatment

MRC-5 cells were cultured in minimum essential medium (Gibco) with 10% fetal calf serum (Gibco) and 1% penicillin–streptomycin. NIH/3T3 cells were grown in Dulbecco’s modified Eagle’s medium (Gibco) with 10% newborn calf serum (Every Green, China) and 1% penicillin–streptomycin instead. All cells were placed at 37 ℃ in a humidified atmosphere with 5% CO_2_. The MRC-5 and NIH/3T3 cells were treated with 5 ng/ml recombinant TGF-β1 (Peprotect, USA) for 48 h to induce their activation. (Data on NIH/3T3 cells are available in the Supplementary Materials.)

MiR-320a-3p mimics, miR-320a-3p inhibitors, siRNA -ALKBH5, and siRNA-FOXM1 were synthesized by Genepharma (Shanghai, China). FOXM1-overexpressed plasmids were purchased from GNEEbay (China). RiboFECT CP reagent (RiboBio Co, Guangzhou, China) was used for cell transfection according to manufacturer instructions. At 24 h after transfection, the fibroblasts were treated with 5 ng/ml TGF-β1 for 48 h.

### Western blot analysis and antibodies

Total protein of lung tissue samples was extracted by using T-PER tissue protein extraction reagent (Thermo Scientific), while total cellular proteins were extracted using Radioimmunoprecipitation Assay (RIPA) lysis buffer (Beyotime, China) and phenylmethylsulfonyl fluoride (Sigma-Aldrich). Protein quantification and sodium dodecyl sulfate (SDS)-polyacrylamide gel electrophoresis (PAGE) were carried out as described previously [29]. Antibodies for collagen I, fibronectin, α-SMA, and ALKBH5 were purchased from Abcam. Antibodies for vimentin and glyceraldehyde-3-phosphate dehydrogenase (GAPDH) were acquired from ABclonal Technology. Anti-FOXM1 was obtained from Santa Cruz.

### Quantitative real-time PCR (qRT-PCR) analysis

RNA isolation was carried out using TRIzol reagent (Tiangen, Beijing, China) according to instructions. Total RNA (500 ng) was reverse transcribed into complementary DNA (cDNA) by using a Bio-Rad T100 thermal cycler (Hercules, CA, USA). qRT-PCR, the amplification reactions were conducted usingith SYBR Green 2 × PCR mix (Vazyme Biotech, Nanjing, China) on a LightCycler 480II (Roche, Switzerland) according to protocols.

### EdU assay

The Cell-Light EdU DNA cell proliferation kit (RiboBio, Guangzhou, China) was used to detect the ability of cell proliferation following each manufacturer’s protocol. Briefly, after incubation with 100 μl 50 μM Edu solution for 2 h, the cells were fixed with 4% carbinol for 30 min (washing cells between each step). Permeabilization buffer was added, followed by incubation for 15 min. Fluorescently labelled EdU was then added to the reaction mixture, followed by incubation for 30 min. Finally, the nuclei were dyed with DAPI and analyzed by using a microscope (Nikon Ti, Tokyo, Japan).

### Immunofluorescence assay

MRC-5 cells fixed with 4% paraformaldehyde were washed three times for 5 min, blocked with 1% goat serum for 1 h at room temperature, incubated with the required antibody, washed three times in PBS, incubated with Cy3-conjugated secondary antibody, washed three times in PBS again, treated with DAPI to dye the nuclei, and finally washed three times in PBS. Images were acquired by Nikon Ti microscope (Tokyo, Japan).

### Dual-luciferase reporter gene assay

The design and synthesis of the FOXM1 reporter plasmids (pGL3-FOXM1 3′UTR-wt and pGL3-FOXM1 3′UTR-mut) were executed by GNEEbay (China). The MRC-5 cells were cultured in 24-well plates and transfected with 400 ng luciferase reporter plasmids together with 50 nM miR-320a-3p mimic/inhibitor/NC. At 24 h after transfection, the dual-luciferase reporter assay kit (Beyotime, China) was used to measure firefly and *Renilla* luciferase activities considering the manufacturer’s protocol.

### Me-RIP assay

The Me-RIP assay was conducted using a Magna RIP RNA-binding protein immunoprecipitation kit (Millipore, Billerica, MA, USA) according to protocols. Briefly, MRC-5 cells were harvested and RNA was extracted to perform me-RIP. Magnetic beads were incubated with rabbit anti-m^6^A monoclonal antibody (Millipore, Temecula, CA, USA) (rabbit IgG antibody served as negative control) at 4 ℃ overnight. Then, beads and RIP immunoprecipitation buffer and fragmented RNAs were mixed well and incubated at 4℃ overnight. The complex was digested with proteinase K buffer. RNA was purified with phenol:chloroform:isoamyl alcohol (125:24:1), followed by reverse transcription and qRT-PCR to detect the enrichment of pri-miR-320a-3p, miR-320a-3p, pre-FOXM1, and FOXM1.

### RIP and pull-down assays

RIP assays were carried out using the Magna RIP RNA-binding protein immunoprecipitation kit (Millipore, Billerica, MA, USA) according to protocols. The RIP method was similar to that used for me-RIP. DGCR8 antibody (Abcam, CA, USA) was used for RIP, and qRT-PCR was performed to detect coprecipitated RNA.

For the RNA pull-down assay, concisely, miR-320a-3p synthesized by RiboBio (Guangzhou, China) and negative control RNA were labeled by using the Pierce RNA 3′-end desthiobiotinylation kit (20163, Thermo Fisher Scientific). The pull-down assay was then carried out using the Thermo Scientific Pierce magnetic RNA–protein pull-down kit (20164, Thermo Fisher Scientific) with its protocol. Briefly, the streptavidin magnetic beads were prewashed twice with 2 × volume of 0.1 M NaOH, 50 mM (nuclease-free) NaCl, and once in 100 mM NaCl. Cell lysates were prepared using standard lysis buffer and incubated with beads with rotation at 4 °C overnight to pull down the RNA complexes. Finally, the RNA-binding complexes were washed and eluted for further qRT-PCR analysis.

### Actinomycin D assay

To block transcription, 1 μg/ml actinomycin D (Gibco) was added to the MRC-5 cell culture medium. After incubation for 2, 4, 6, or 12 h, the cells were harvested and RNA was extracted to detect the stability of FOXM1 using qRT-PCR.

### Statistical analysis

All experiments were repeated at least three times, and data are shown as mean ± standard deviation (SD). Independent-samples *t*-test and one-way analysis of variance (ANOVA) were followed by Tukey’s test for > 2-group comparisons as indicated in the manuscript. *P* < 0.05 was considered significant. GraphPad Prism 6.01 was used to draw the figures.

## Results

### ALKBH5 was upregulated in both lung tissues from the silica-inhaled mouse model and TGF-β1-activated lung fibroblasts, and knockdown of ALKBH5 inhibited TGF-β1-induced fibroblast activation

**Fig. 1:**
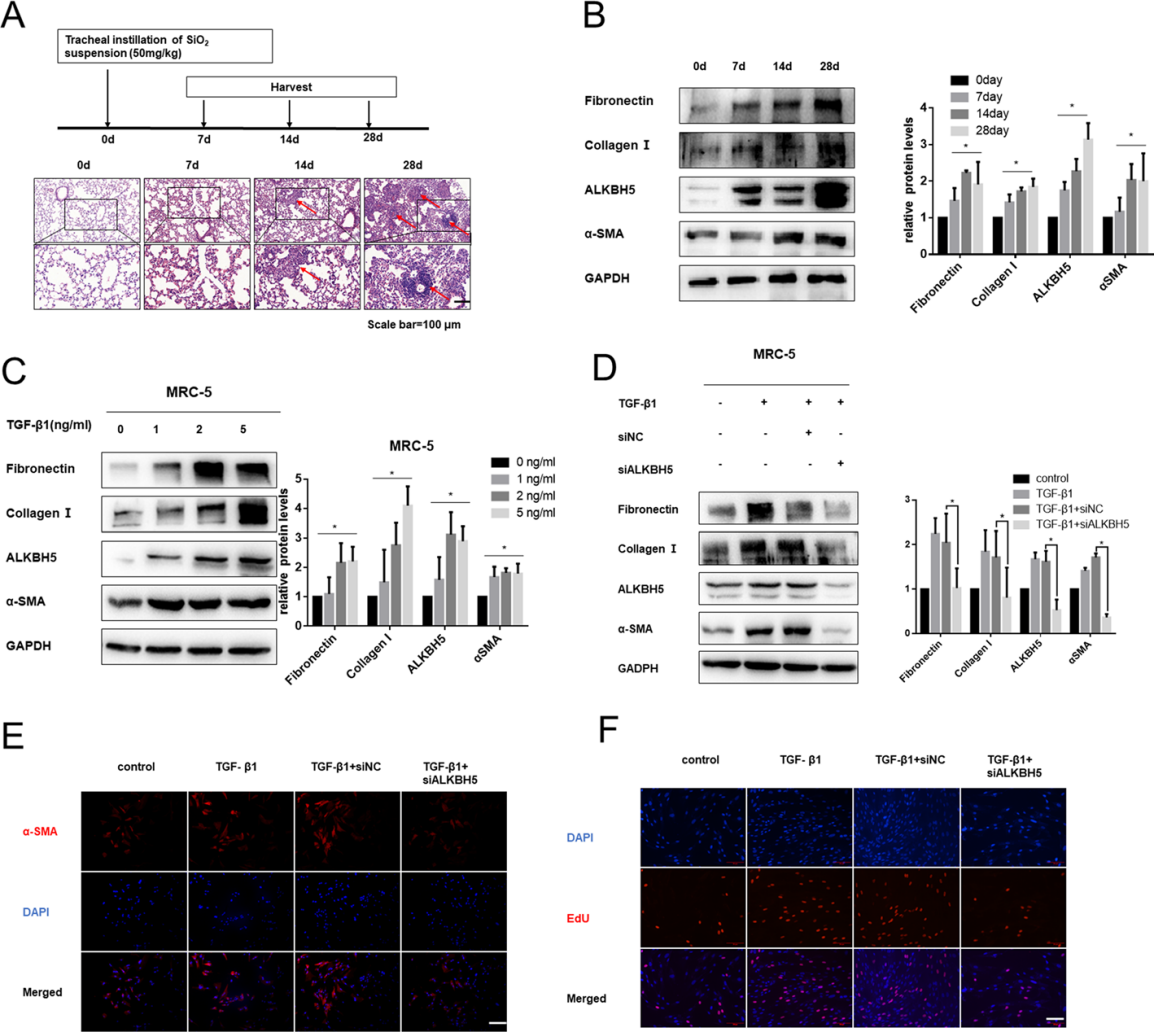
ALKBH5 was upregulated in silica-induced pulmonary fibrosis and knockdown of ALKBH5 inhibited TGF-β-induced fibroblast activation. **A** Pathological changes in mouse lung tissue presented by H&E staining; arrow indicates representative fibrosis foci (*n* = 6 for each group). **B** Western blotting analysis of fibronectin, collagen I, ALKBH5, and α-SMA in each group. The results of the experiment were repeated at least three times. **C** MRC-5 cells were treated with 0, 1, 2, and 5 ng/ml TGF-β1 for 48 h, and protein levels of fibronectin, collagen I, ALKBH5, and α-SMA were examined by the western bolt. The results of the experiment were repeated at least three times. **D** Western blotting analysis of the relative protein levels of MRC-5 cells transfected with 50 nM siNC or siALKBH5 before treatment with 5 ng/mL TGF-β1 for 48 h. The results of the experiment were repeated at least three times. **E** Immunofluorescence staining detected α-SMA (red) levels in different groups; DNA staining by DAPI (blue) represents nuclei; bars = 100 μm. **F** EdU staining for assessment of cell proliferation in MRC-5 cells, showing that silencing of ALKBH5 inhibited TGF-β1-induced cell viability and proliferation; bars = 100 μm. Data expressed as mean ± SD of at least three independent experiments, *p < 0.05

We characterized the expression pattern of the critical demethylase ALKBH5 during silica-induced pulmonary fibrosis. Firstly, we established the silica-induced pulmonary mouse model as described above. H&E staining analysis showed destruction of alveolar structure and typical fibrotic nodules on day 28 after silica instillation (Fig. 1A). The protein levels of mesenchymal cell marker (α-SMA) and main components of the extracellular matrix (fibronectin and collagen I) were prominently increased (Fig. 1B). Hydroxyproline content assay (Additional file 1: Fig. S1A) further verified the success of the mouse lung fibrosis model. More importantly, the expression of ALKBH5 was significantly increased (Fig. 1B). To further interrogate our results, we examined ALKBH5 expression during the activation of fibroblasts, the principal cells responsible for pulmonary fibrosis. MRC-5 and NIH/3T3 cell lines were subjected to TGF-β1-induced activation and transdifferentiation from a quiescent to myofibroblast phenotype (Fig. 1C, Additional file 1: Fig. S1D). ALKBH5 was significantly upregulated and increased in a dose-dependent manner when treated with TGF-β1 (0, 1, 2, and 5 ng/ml) for 48 h (Fig. 1C; Additional file 1: Fig. S1B-D), suggesting that ALKBH5 may be associated with the silica-induced pulmonary fibrosis process. We chose 5 ng/ml TGF-β1 to treat cells for 48 h in the subsequent experiments.

To determine whether ALKBH5 plays a role in TGF-β1-induced fibroblast activation, we used siRNA to knock down ALKBH5 (siALKBH5). Compared with a negative control siRNA, the siALKBH5 significantly reduced ALKBH5 expression (Fig. 1D; Additional file 1: Fig. S1E-S1G). Western blot analysis showed that ALKBH5 knockdown led to inhibition of production of fibrotic markers (Fig. 1D; Additional file 1: Fig. S1G), as demonstrated by immunofluorescence with the typical myofibroblast makers alpha-smooth muscle actin (α-SMA) (Fig. 1E). Moreover, we found that treatment with TGF-β1 increased the cell proliferative ability; depletion of ALKBH5, however, inhibited cell proliferation, as shown by EdU fluorescence staining (Fig. 1F).

### ALKBH5 regulated processing of miR-320a-3p by DGCR8 in an m^6^A-dependent manner

**Fig. 2:**
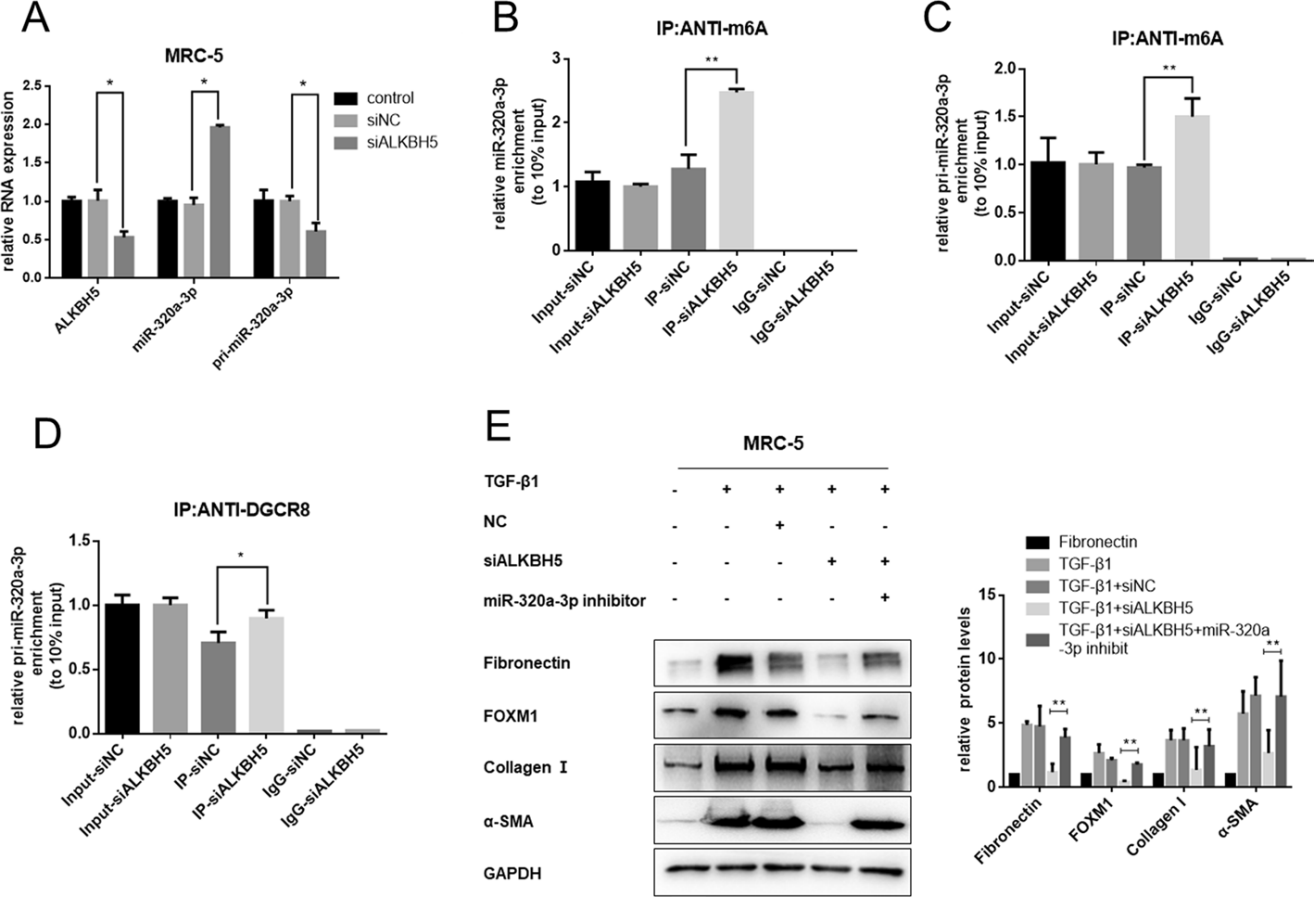
ALKBH5 regulates processing of miR-320a-3p by DGCR8 in an m^6^A-dependent manner to have an antifibrotic effect. **A** Expression of ALKBH5, miR-320a-3p, and pri-miR-320a-3p in MRC-5 cells after transfection with siNC or siALKBH5 by qRT-PCR analysis with U6 as internal reference. Detection of miR-320a-3p (**B**) and pri-miR-320a-3p (**C**) m^6^A modification levels by RIP of m^6^A-modified miRNA in siNC or siALKBH5 groups followed by qRT-PCR. **D** Detection of pri-miR-320a-3p binding to DGCR8 by immunoprecipitation of DGCR8-associated RNA from MRC-5 cells with transfected siNC or siALKBH5 followed by qRT-PCR. **E** After co-transfection with siALKBH5 and miR-320a-3p inhibitor, MRC-5 cells were administrated 5 ng/ml TGF-β1 for 48 h. Western blotting showed fibronectin, collagen I, FOXM1, and α-SMA protein levels. All data expressed as mean ± SD of at least three independent experiments, *p < 0.05 and **p < 0.01

Previously, Alarcon et al. found that the microprocessor protein DGCR8 could recognize and positively modulate the methylated pri-microRNA process [16]. Human RNA helicase DDX3 could interact with ALKBH5 and modulate the demethylation of mRNAs and miRNAs [27]. Our previous miRNA microarray of silica-induced mouse lung tissues showed that various miRNAs were dysregulated [29], and downregulation of miR-320a-3p was significant (Additional file 3: Fig. S3A). Thus, we assessed whether ALKBH5 was required for pri-miR-320a-3p processing in fibroblast activation during silica-induced pulmonary fibrosis. The qRT-PCR results showed that miR-320a-3p was statistically increased in ALKBH5-knockdown cells, while expression of pri-miR-320a-3p was decreased (Fig. 2A; Additional file 2: Fig. S2A). Notably, me-RIP revealed that ALKBH5 knockdown increased the amount of miR-320a-3p and pri-miR-320a-3p modified by m^6^A (Fig. 2B, C). We found an increased level of pri-miR-320a-3p binding by DGCR8 immunoprecipitated from knockdown ALKBH5 cells (Fig. 2D). These results suggest that knockdown ALKBH5 could enhance the recognition of pri-miR-320a-3p by DGCR8 and promote miR-320a-3p maturation in an m^6^A-dependent manner.

To detect whether ALKBH5 could inhibit TGF-β1-induced fibroblast activation by regulating the processing of miR-320a-3p, we cotransfected siALKBH5 and miR-320a-3p inhibitors. Inhibition of miR-320a-3p expression partly reversed the ALKBH5-knockdown-induced antifibrotic effect (Fig. 2E; Additional file 2: Fig. S2B).

### miR-320a-3p could be involved in the pathogenesis of silica-induced pulmonary fibrosis

**Fig. 3:**
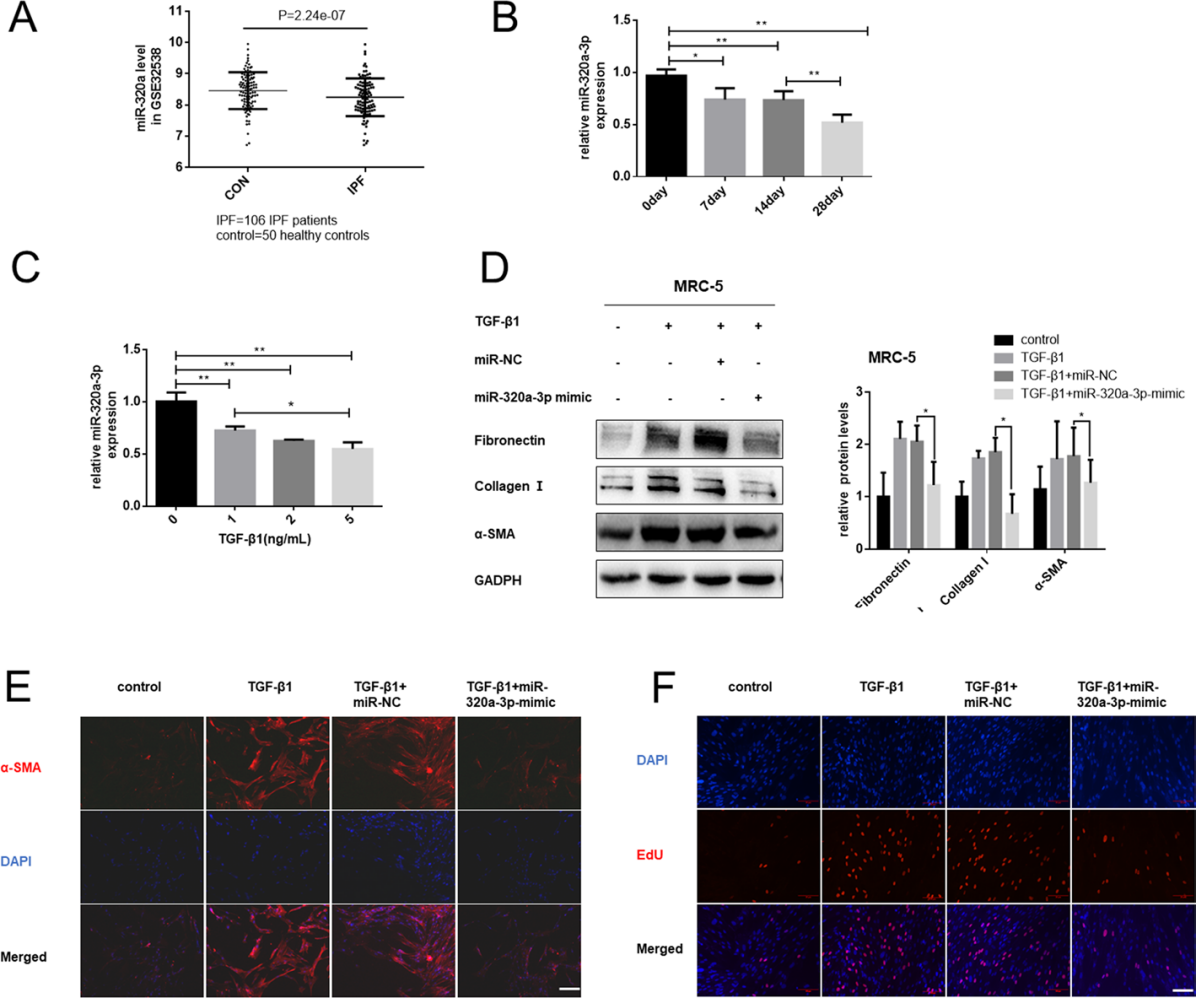
miR-320a-3p could be involved in the pathogenic mechanism of silica-induced pulmonary fibrosis. **A** Relative expression of miR-320a-3p in 50 healthy controls and 106 IPF patients from an IPF database (GSE32538); *n* = 106 in IPF group, and *n* = 50 in control group. **B** qRT-PCR analysis of miR-320a-3p expression in mouse fibrotic lung tissues on days 7, 14, and 28 (*n* = 6 for each group). **C** miR-320a-3p was downregulated in TGF-β1-induced fibroblast activation, as evaluated by qRT-PCR. **D** Western blotting analysis of relative protein levels of MRC-5 cells transfected with 20 nM miR-NC or miR-320a-3p mimics before treatment with 5 ng/mL TGF-β1 for 48 h. **E** Immunofluorescence staining detected α-SMA (red) levels in different groups; DNA staining by DAPI (blue) represents nuclei; bars = 100 μm. **F** EdU staining for assessment of cell proliferation in MRC-5 cells, showing that overexpression of miR-320a-3p reversed TGF-β1-induced cell viability and proliferation; bars = 100 μm. All data expressed as mean ± SD of at least three independent experiments, *p < 0.05 and **p < 0.01

We observed significant downregulation of miR-320a-3p in IPF patients in an IPF database (GSE32538) (Fig. 3A), and our previous miRNA microarray also showed that miR-320a-3p was decreased (Additional file 3: Fig. S3A). Similarly, we found that miR-320a-3p was downregulated in silica-induced mouse lung tissue and TGF-β1-treated fibroblasts (Fig. 3B, C), indicating that miR-320a-3p could be involved in the fibrosis process.

Having verified the expression model of miR-320a-3p in pulmonary fibrosis, we explored whether restoring miR-320a-3p could alleviate the process of TGF-β1-induced fibroblast activation. As shown by qRT-PCR assay, transfected miR-320a-3p mimic statistically increased its expression (Additional file 3: Fig. S3D, E). Moreover, overexpression of miR-320a-3p inhibited upregulation of TGF-β1-induced fibrosis markers, as well as the enhancement of cell viability and proliferation (Fig. 3D–F; Additional file 3: Fig. S3F).

### FOXM1 is the direct downstream target of miR-320a-3p

**Fig. 4:**
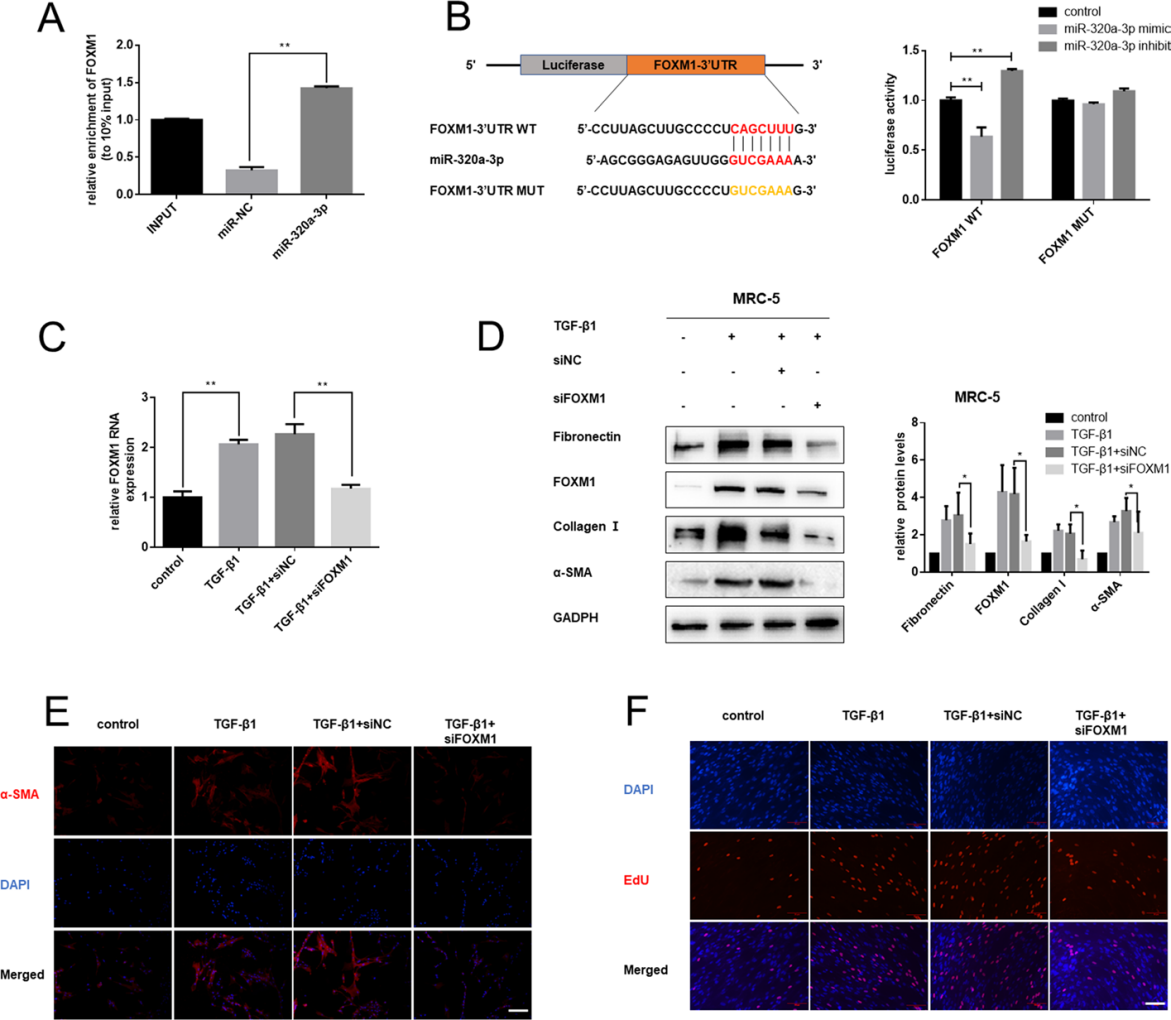
FOXM1 is the direct and functional target of miR-320a-3p. **A** The interaction between miR-320a-3p and FOXM1 mRNA was verified by RNA pull-down assay. **B** Dual-luciferase reporter assay verified FOXM1 as the downstream target of miR-320a-3p. **C** Transfection of siFOXM1 significantly decreased FOXM1 mRNA expression in MRC-5 cells treated with 5 ng/mL TGF-β1 for 48 h. Western blot (**D**) and immunofluorescence staining (**E**) analysis revealed that FOXM1 knockdown alleviated TGF-β1-induced fibrotic protein expression; bars = 100 μm. **F** EdU staining for assessment of cell proliferation in MRC-5 cells, showing that FOXM1 inhibition reversed TGF-β1-induced cell viability and proliferation; bars = 100 μm. All data expressed as mean ± SD of at least three independent experiments, *p < 0.05 and **p < 0.01

To clarify the potential molecular mechanism of miR-320a-3p, we used prediction algorithms, including TargetScan and StarBase, and identified FOXM1 as a potential target of miR-320a-3p. FOXM1, which was identified as a critical driver of lung fibroblast activation and fibrogenesis [30], had a presumptive binding site of miR-320a-3p in 3′-untranslated regions (UTR). Consistently, a pull-down assay showed that miR-320a-3p could interact with FOXM1 (Fig. 4A). To further verify the direct interaction between miR-320a-3p and FOXM1, a dual-luciferase reporter gene assay was performed in MRC-5 cells. The wild-type 3′-UTR sequence and the mutant 3′-UTR sequence of FOXM1 were cloned to construct reporter plasmids, respectively. The result showed that cotransfection of miR-320a-3p mimic/inhibitor and wild-type reporter gene plasmid decreased/increased the luciferase activity significantly but not the mutant reporter (Fig. 4B).

We next sought to determine whether FOXM1 correlates with the process of pulmonary fibrosis. The results showed that FOXM1 was remarkably upregulated in TGF-β1-treated fibroblasts (Additional file 4: Fig. S4A–C). To further elucidate the specific mechanisms, we knocked down FOXM1 expression using siFOXM1 and confirmed the knockdown efficiency (Fig. 4C; Additional file 4: Fig. S4D). Also, we found that knockdown of FOXM1 inhibited expression of fibrosis markers (Fig. 4D; Additional file 4: Fig. S4E). Moreover, depletion of FOXM1 reversed the abnormal elevation of cell viability and proliferation induced by TGF-β1 (Fig. 4E, F). Together, these results suggest that miR-320a-3p could target FOXM1, and the latter could be involved in the TGF-β1-induced activation of fibroblasts.

### Overexpression of miR-320a-3p exerted antifibrotic effects both in vitro and in vivo by targeting FOXM1

**Fig. 5:**
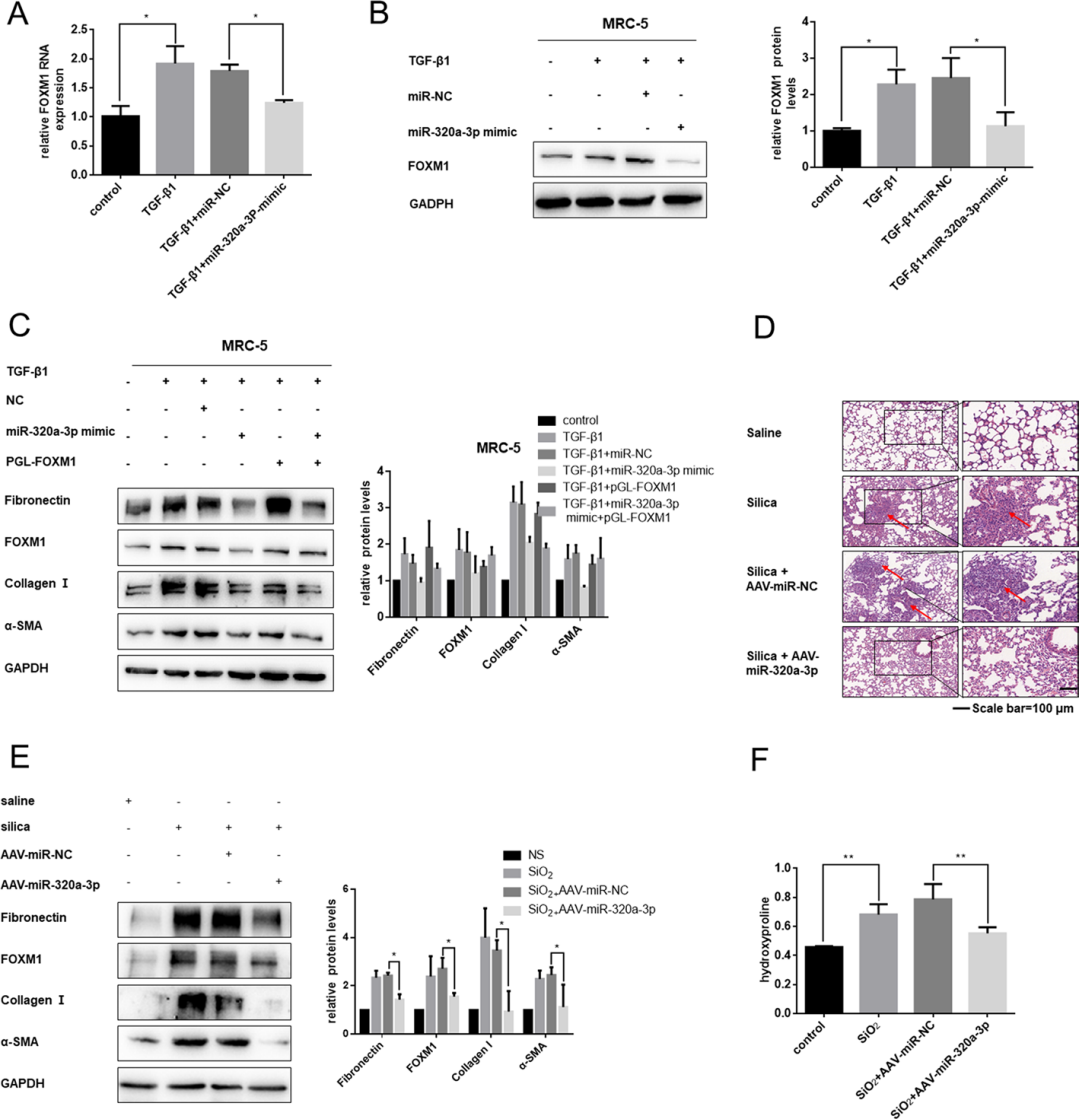
Overexpression of miR-320a-3p exerts antifibrotic effects both in vitro and in vivo by targeting FOXM1. **A** qRT-PCR and **B** western blot analysis showing that enhanced miR-320a-3p expression inhibited FOXM1 expression induced by TGF-β1. **C** Western bolt showing that overexpression of FOXM1 significantly counteracted the inhibitory effects of miR-320a-3p in fibroblast activation via TGF-β1 treatment. **D** Sections stained with H&E suggested the lung fibrotic lesion of each group; arrow indicates representative fibrosis foci (*n* = 6 in each group). **E** Western blot analysis of fibronectin, collagen I, FOXM1, and α-SMA in each group. **F** Collagen deposition was detected by hydroxyproline content assay. All data expressed as mean ± SD of at least three independent experiments, *p < 0.05 and **p < 0.01

Next, we investigated whether overexpression of miR-320a-3p could suppress TGF-β1-induced activation of fibroblasts by targeting FOXM1. In vitro, miR-320a-3p mimic inhibited FOXM1 expression at both mRNA and protein levels (Fig. 5A, B; Additional file 5: Fig. S5A, B). Overexpression of FOXM1 could abrogate miR-320a-3p-alleviated fibroblast activation (Fig. 5C; Additional file 5: Fig. S5C).

To better interrupt the intervention effects of miR-320a-3p in silica-induced pulmonary fibrosis, we also overexpressed miR-320a-3p in the silica-induced mouse pulmonary fibrosis model. As expected, increased miR-320a-3p did alleviate the inflammation and destruction of alveolar architecture (Fig. 5D). Consistent with this, we found evident improvement in the severity and location of fibrotic lesions (Additional file 6: Table S1). FOXM1 was decreased at both mRNA and protein levels (Fig. 5E; Additional file 5: Fig. S5D, E). Enhanced expression of miR-320a-3p inhibited silica-induced fibrogenesis and collagen production in mouse lungs (Fig. 5E, F). In general, these results showed the antifibrotic effect of miR-320a-3p in silica-induced pulmonary fibrosis by targeting FOXM1.

### FOXM1 is also regulated by ALKBH5 directly in an m^6^A-dependent manner

**Fig. 6:**
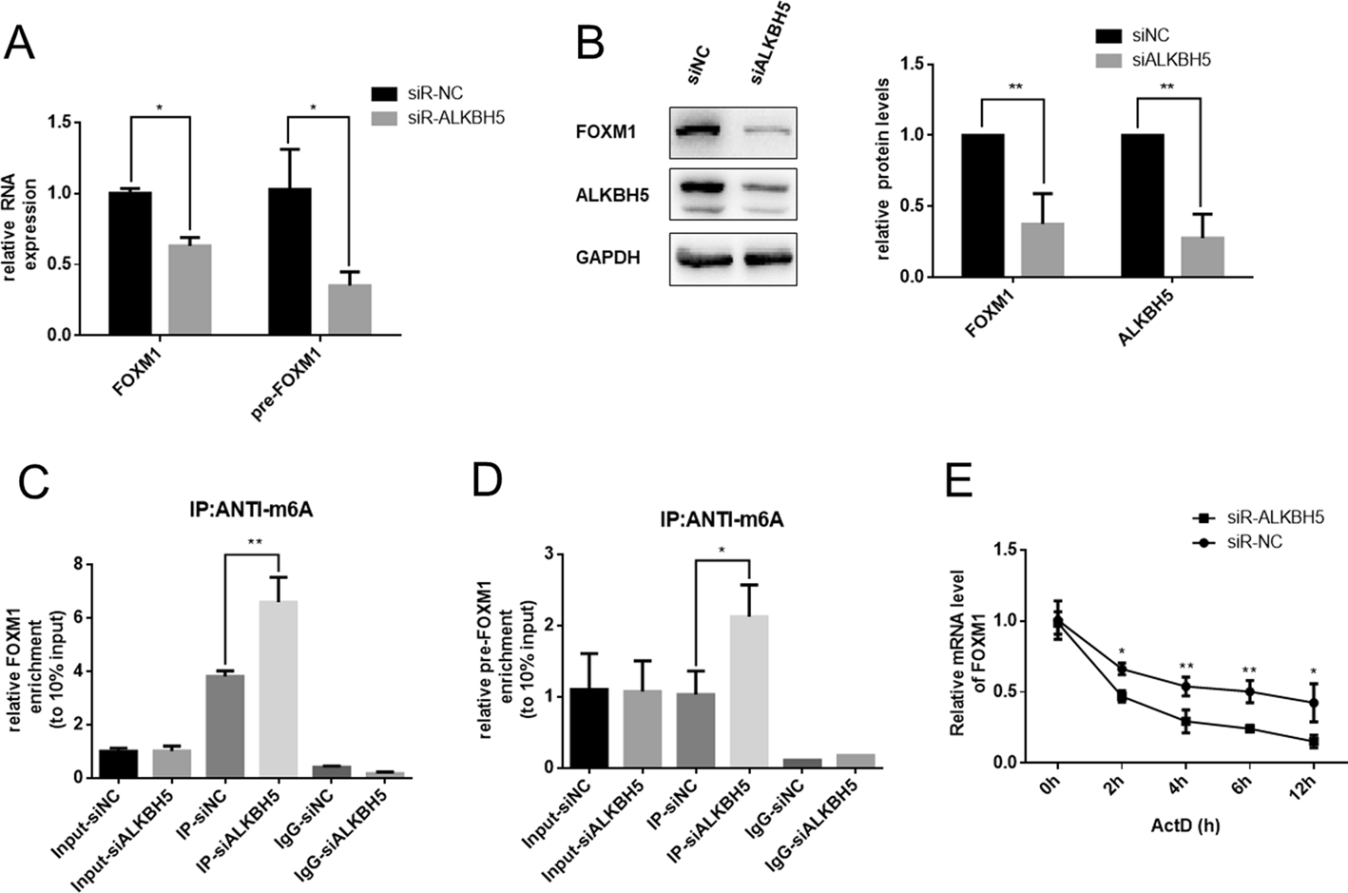
FOXM1 is also regulated by ALKBH5 directly in an m^6^A-dependent manner. **A** Knockdown of ALKBH5 inhibited both FOXM1 mRNA and pre-mRNA expression, as showed by qRT-PCR analysis. **B** Western blot analysis showed that inhibition of ALKBH5 decreased FOXM1 protein expression. **C** and **D** Detection of FOXM1 mRNA and FOXM1 pre-mRNA m^6^A modification levels by immunoprecipitation of m^6^A-modified miRNA in siNC or siALKBH5 groups followed by qRT-PCR. **E** mRNA level of FOXM1 analyzed by qRT-PCR in actinomycin D-treated MRC-5 cells with transfected siNC or siALKBH5. All data expressed as mean ± SD of at least three independent experiments, *p < 0.05 and **p < 0.01

**Fig. 7:**
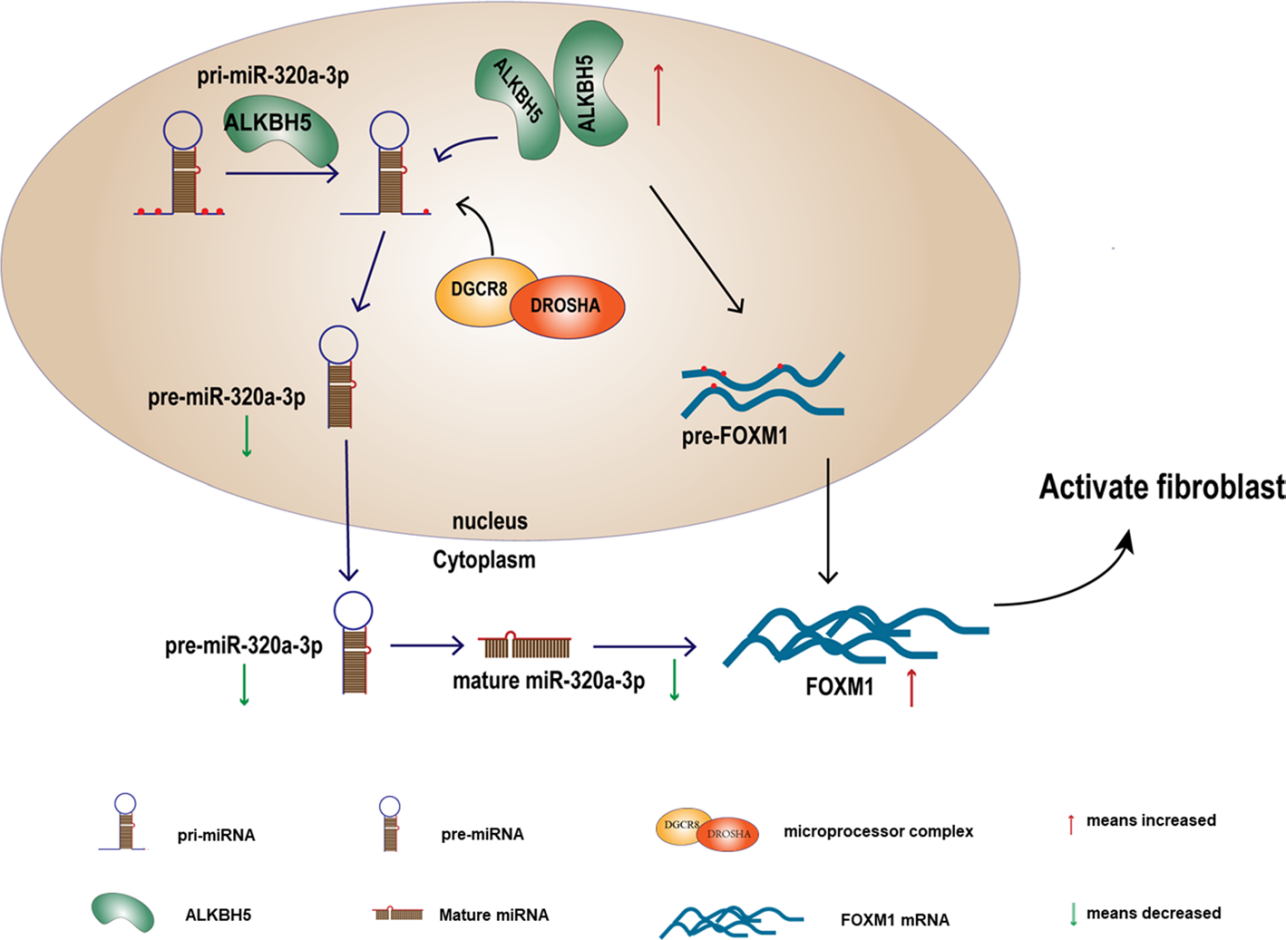
Mode pattern of ALKBH5/miR-320a-3p/FOXM1 and ALKBH5/FOXM1 regulatory network in silica-induced pulmonary fibrosis

Previously, Zhang et al. [23] found that ALKBH5 could interact with the FOXM1 3′-UTR region and sustain FOXM1 expression through its demethylation activity. So, we also assessed whether ALKBH5 could regulate FOXM1 expression in TGF-β1-treated fibroblasts. Firstly, we observed that FOXM1 mRNA and pre-mRNA were decreased significantly in ALKBH5-knockdown cells (Fig. 6A) as well as protein levels (Fig. 6B). Moreover, MeRIP revealed that ALKBH5 knockdown significantly increased FOXM1 mRNA and pre-mRNA modified by m^6^A (Fig. 6C, D). The best-established function for m^6^A, which was identified in 1978, causes mRNA instability [31]. Consistently, we analyzed FOXM1 mRNA degradation by qRT-PCR in actinomycin D (ActD, transcription inhibitor)-treated MRC-5 cells. Compared with control vectors, the decay of FOXM1 mRNA was increased in siALKBH5-transfected MRC-5 (Fig. 6E). These observations indicated that ALKBH5 could demethylate FOXM1 pre-mRNA and mRNA and elevate expression of FOXM1 (Fig. 7).

## Discussion

Silica-induced fibrosis remains a severe public health problem. In 2022, more than 4400 clinical studies were listed on clinicaltrials.gov with the keyword “fibrosis,” of which over 800 were lung fibrosis. Despite these great efforts, lung transplant is often the only available option for patients with severe pulmonary fibrosis. The difficulty of curing fibrosis has many reasons, among which the main causes are that the related mechanisms have not been fully uncovered [32]. In the current study, we clarified the molecular mechanism of m^6^A modification and function of miR-320a-3p and FOXM1, regulated by ALKBH5 in TGF-β1-induced activated fibroblasts. The present findings indicate that m^6^A modification regulated by ALKBH5 is a novel target for potential lung fibrosis therapy.

*N*^6^-methyladenosine (m^6^A) methylation is one of the most common and reversible RNA modifications in eukaryotes, executing essential functions in normal life activities and diseases [33]. Demethylase acts as an “eraser” composed of FTO and ALKBH5. Recently, ALKBH5 has aroused significant biological and pharmacological interest among researchers [22]. A growing number of studies have demonstrated that ALKBH5 is involved in various diseases [34], such as lung cancer [35]. However, in the field of cancer research, the potential mechanisms of ALKBH5 are not only unclear but also disputed. The reason for the controversial function of ALKBH5 in cancer likely relates to the communication between ALKBH5 with long coding RNAs, miRNAs, mRNAs, etc. [24, 26, 36, 37]. Despite the many cancer and noncaner studies about ALKBH5, the importance of ALKBH5 expression in silica-induced pulmonary fibrosis remains unclear. The current findings show that ALKBH5 was upregulated in, and that its knockdown inhibited, TGF-β1-induced fibroblast activation.

Further study revealed that ALKBH5 decreased expression of miR-320a-3p. Previous studies have shown that m^6^A methylation is an important regulatory factor in miRNA maturation [16, 38]. For example, it has been demonstrated that METTL3 can promote the maturity of miR-221/222 [39] and miR-126B [16] by binding with DGCR8, which is a pivotal protein underlying pri-miRNA processing. ALKBH5 was also reported to inhibit autophagy of epithelial ovarian cancer through miR-7 and BCL-2 [24]. Besides, it was reported recently that ALKBH5 could inhibit miR-107/LATS2-mediated YAP activity by inhibiting tumor growth in non-small cell lung cancer [26]. These studies suggest a critical role of ALKBH5 in miRNA regulation, although the exact molecular mechanism is still not clear. As shown in the current study, ALKBH5 could eliminate the m^6^A mark in pri-miR-320a-3p to inhibit pri-miRNA processing. Our result is consistent with a previous study that verified that DDX3 interacted with ALKBH5, regulating microRNAs’ methylation status [27]. On this basis, our study further explored the effect of ALKBH5 on miRNA functions by regulating microRNAs’ methylation status. However, in silica-induced pulmonary fibrosis, whether DDX3 is required for ALKBH5 to regulate the methylation status of miR-320a-3p and FOXM1 needs to be further investigated. More importantly, this may be an underlying mechanism for the diversity of ALKBH5 regulatory patterns. On the other hand, it is also indicated that m^6^A in miRNA is a dynamic and reversible modification as in mRNA [40]. Notably, we observed that ALKBH5 knockdown increased the m^6^A level of mature miR-320a-3p. Previous study has shown that RNA modification may also exist in mature miRNAs [17]. They found upregulation of methylated miRNAs in gastrointestinal cancer; however, m^6^A methylation in mature miRNAs causes a large structural change and affects the target RNA recognition, which contradicts our results. It was not unusual for vital regulatory factors to be regulated in multiple ways in different pathological and physiological states [41, 42]. In the case of our study, the processing regulation of miR-320a-3p may either precede or dominate over structural change. Nevertheless, further studies are still needed to clarify whether methylation modifications in mature miR-320a-3p alter its stability and target recognition in silica-induced pulmonary fibrosis.

FOXM1 was described as an exclusively proliferation-specific mammalian transcription factor [43], but subsequent studies demonstrated that it also controls cell cycle progression, migration, invasion, angiogenesis, metastasis, and other physiological and pathological processes [44]. Intriguingly, emerging evidence indicates that FOXM1 plays a key role in lung diseases [30, 45]. Previous investigation showed that FOXM1 was more highly expressed in bleomycin-induced mouse lung fibrosis, and deletion of FOXM1 attenuated the pulmonary fibrosis [30]. Consistent with these previous findings, our results showed that FOXM1 was upregulated in both silica-induced murine fibrotic lungs and TGF-β1-treated fibroblasts. Our work further confirmed that FOXM1 is the direct and functional target of miR-3p, and finally revealed the ALKBH5/miR-320q-3p/FOXM1 axis in lung fibroblasts activation during silica-induced lung fibrosis.

ALKBH5 has been verified to demethylate FOXM1 nascent transcripts by the FOXM1 3′-UTR region, resulting in enhanced FOXM1 expression in glioblastoma stem-like cells [23]. In head and neck squamous cell carcinoma, human RNA helicase DDX3 could modulate cisplatin resistance via ALKBH5-mediated m^6^A demethylation of FOXM1 and NANOG [46]. The ALKBH5-m^6^A–FOXM1 signaling axis has also been reported to promote proliferation and invasion of lung adenocarcinoma cells [36]. Therefore, the ALKBH5-m^6^A–FOXM1 signaling axis attracted our interest, and we validated that ALKBH5 could regulate FOXM1 mRNA stability via demethylation.

Together, these findings put the spotlight on the ALKBH5-(miR-320a-3p)-FOXM1 axis as a potential target for antifibrotic strategies. Our study is not free of limitations; for example, although we verified the upregulation of ALKBH5 in silica-induced mouse pulmonary fibrosis tissues, the intervention effect remains unclarified in vivo and must be addressed in further investigations. Nonetheless, adding to the increasing importance of RNA posttranscriptional modification in normal physiological processes, the current investigation reveals a key role for m^6^A in silica-induced pulmonary fibrosis. This is significant because there is an imperative demand for the development of new antifibrotic drugs [3, 4, 47]. Our data provide a proof of concept that targeting ALKBH5 may be efficacious in treating lung fibrosis. It is hoped that some ALKBH5 inhibitors will make it into the clinic shortly.

## Conclusions

The present findings suggest that ALKBH5 promotes silica-induced lung fibrosis via the miR-320a-3p/FOXM1 axis or by targeting FOXM1 directly in an m^6^A-dependent manner. Approaches aimed at ALKBH5 may be efficacious in treating lung fibrosis.

## Supplementary Information

**Additional file 1: Fig. S1.** ALKBH5 is upregulated in silica-induced pulmonary fibrosis, and knockdown of ALKB5 inhibits TGF-β-induced fibroblast activation.

**Additional file 2: Fig. S2.** ALKBH5 regulates processing of miR-320a-3p by DGCR8 in an m^6^A-dependent manner to play an antifibrotic effect.

**Additional file 3: Fig. S3.** miR-320a-3p is involved in the pathogenesis of silica-induced pulmonary fibrosis.

**Additional file 4: Fig. S4.** FOXM1 is the direct and functional target of miR-320a-3p.

**Additional file 5: Fig. S5.** Overexpression of miR-320a-3p exerts antifibrotic effects both in vitro and in vivo by targeting FOXM1.

**Additional file 6: Table S1.** Histologic scores for the severity and distribution of lung lesions.

**Additional file 7.** Original images for western blots.

## Data Availability

The data from this study are available in this published article.

## Abbreviations

ALKBH5: AlkB homolog 5
FOXM1: Forkhead box protein M1
m^6^A: *N*^6^-methyladenosine
DGCR8: RNA‐binding protein DiGeorge syndrome critical region gene 8
RIP: RNA immunoprecipitation
TGF-β1: Transforming growth factor-β1
α-SMA: Alpha-smooth muscle actin
ECM: Extracellular matrix
miRNA: MicroRNA
pri-miRNA: Primary miRNA
qRT-PCR: Quantitative real-time PCR
siRNA: Small interfering RNA
DDX3: Asp-Glu-Ala-Asp (DEAD)-box polypeptide 3
IPF: Idiopathic pulmonary fibrosis
UTR: Untranslated regions
ActD: Actinomycin D
siNC: Small interfering RNA of negative control
siALKBH5/siFOXM1: SiRNA of ALKBH5/FOXM1
pre-RNA: Precursor RNA
H&E: Hematoxylin and eosin
DAPI: 4′,6-Diamidino-2-phenylindole
