# Supplementary material for: ALKBH5 promotes lung fibroblast activation and silica-induced pulmonary fibrosis through miR-320a-3p and FOXM1
Source: Cell Mol Biol Lett. 2022 Mar 12;27:26. doi: 10.1186/s11658-022-00329-5 (PMC8917683; doi:10.1186/s11658-022-00329-5)
Supplement: Supplementary file 1 — Additional file 1: Fig. S1. ALKBH5 is upregulated in silica-induced pulmonary fibrosis, and knockdown of ALKB5 inhibits TGF-β-induced fibroblast activation. [file 11658_2022_329_MOESM1_ESM.docx]

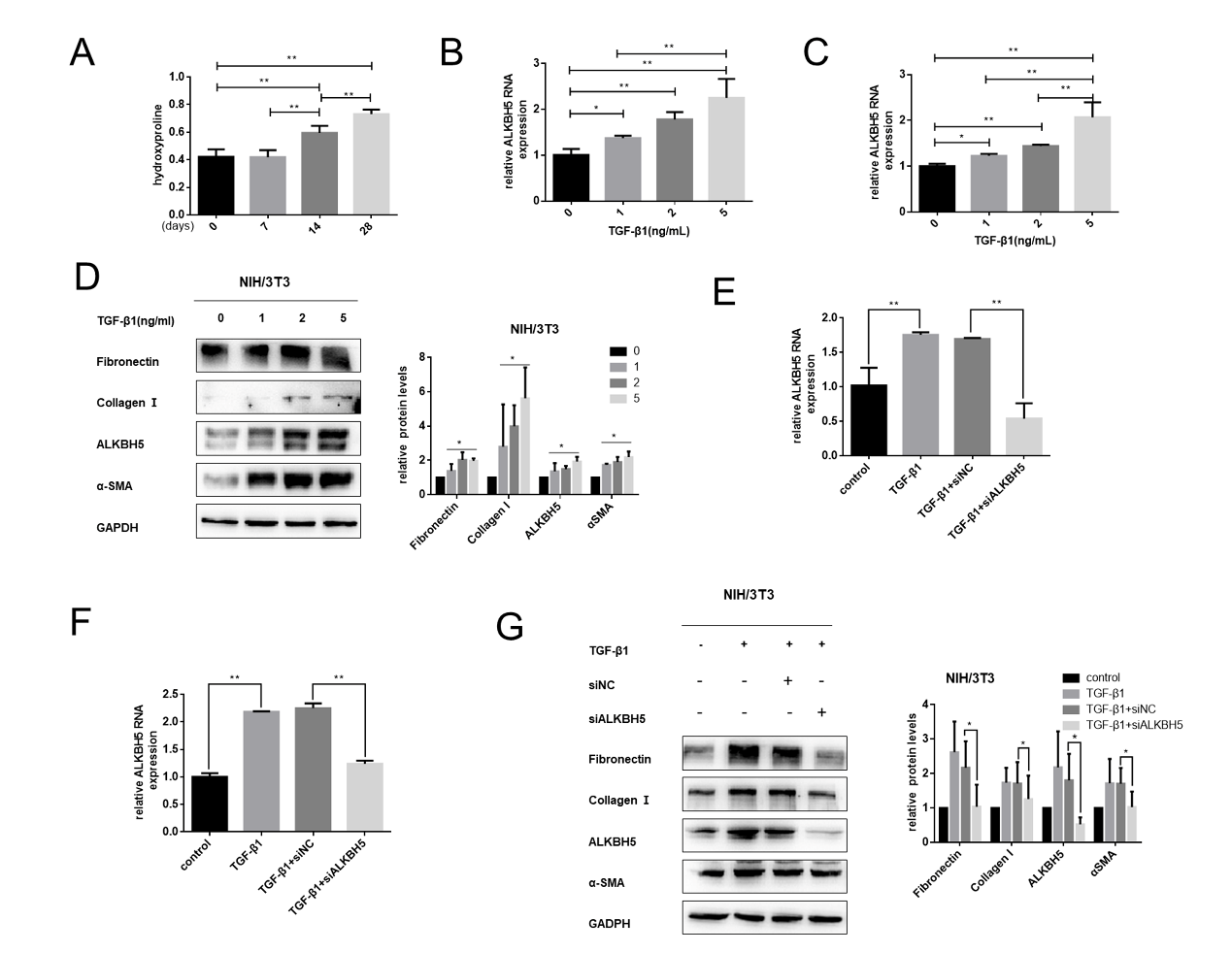


**Figure S1. ALKBH5 is upregulated in silica-induced pulmonary fibrosis and knockdown of ALKB5 inhibits TGF-β-induced fibroblast activation**

(A) The collagen deposition was detected by hydroxyproline content assay, and n = 6 in each group. (B) and (C) Total RNA of MRC-5 and NIH/3T3 cells treated with 0, 1, 2, and 5 ng/ml TGF-β1 were extracted, and relative ALKBH5 expression levels were determined by qRT-PCR; GAPAH mRNA served as internal control. (D) NIH/3T3 cells treated with 0, 1, 2, and 5 ng/ml TGF-β1 for 48h, and protein levels of fibronectin, collagen Ⅰ, ALKBH5, and α-SMA were examined by western bolt. (E) and (F) qRT-PCR detection of ALKBH5 mRNA expression in MRC-5 and NIH/3T3 cells transfected with siNC or siALKBH5 then treated with 5ng/ml TGF-β1 for 48h. (G) Western blotting analysis the relative protein levels of NIH/3T3 cells transfected with 50 nM of siNC or siALKBH5 before treated with 5 ng/mL of TGF-β1 for 48 h. All data were expressed as the means ± SD of at least 3 independent experiments, **p* < 0.05 and ***p* < 0.01.
