# Supplementary material for: ALKBH5 promotes lung fibroblast activation and silica-induced pulmonary fibrosis through miR-320a-3p and FOXM1
Source: Cell Mol Biol Lett. 2022 Mar 12;27:26. doi: 10.1186/s11658-022-00329-5 (PMC8917683; doi:10.1186/s11658-022-00329-5)
Supplement: Supplementary file 2 — Additional file 2: Fig. S2. ALKBH5 regulates processing of miR-320a-3p by DGCR8 in an m6A-dependent manner to play an antifibrotic effect. [file 11658_2022_329_MOESM2_ESM.docx]

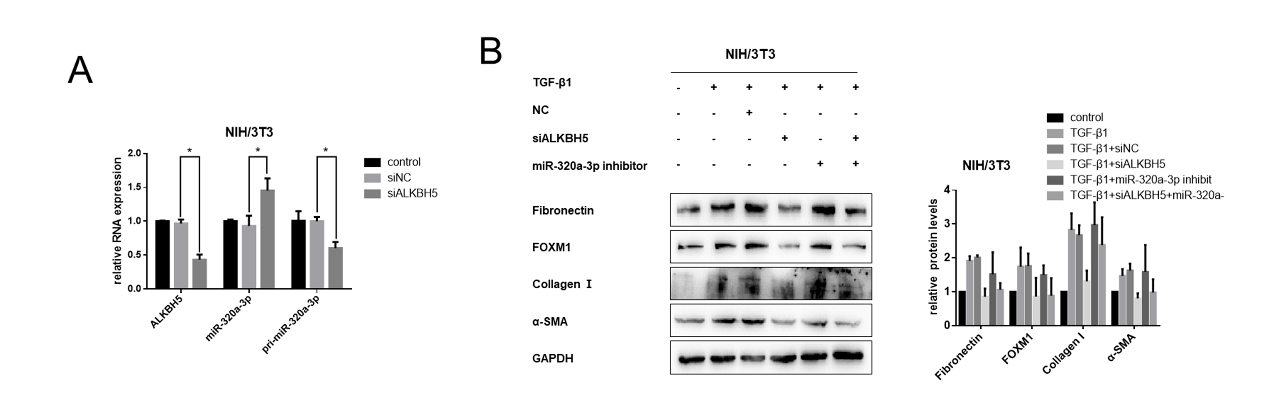


**Figure S2. ALKBH5 regulates the processing of miR-320a-3p by DGCR8 in an m6A manner to play an antifibrotic effect**

(A) The expression of ALKBH5, miR-320a-3p and pri-miR-320a-3p in NIH/3T3 cells after transfected with siNC or siALKBH5 using qRT-PCR analysis; U6 was used as the interval reference. (B) After co-transfected with siALKBH5 and miR-320a-3p inhibitor, NIH/3T3 cells were administrated 5ng/ml TGF-β1 for 48h. Western blotting showing fibronectin, collagen Ⅰ, FOXM1, and α-SMA protein levels. All data were expressed as the means ± SD of at least 3 independent experiments, **p* < 0.05 and ***p* < 0.01.
