# Supplementary material for: ALKBH5 promotes lung fibroblast activation and silica-induced pulmonary fibrosis through miR-320a-3p and FOXM1
Source: Cell Mol Biol Lett. 2022 Mar 12;27:26. doi: 10.1186/s11658-022-00329-5 (PMC8917683; doi:10.1186/s11658-022-00329-5)
Supplement: Supplementary file 3 — Additional file 3: Fig. S3. miR-320a-3p is involved in the pathogenesis of silica-induced pulmonary fibrosis. [file 11658_2022_329_MOESM3_ESM.docx]

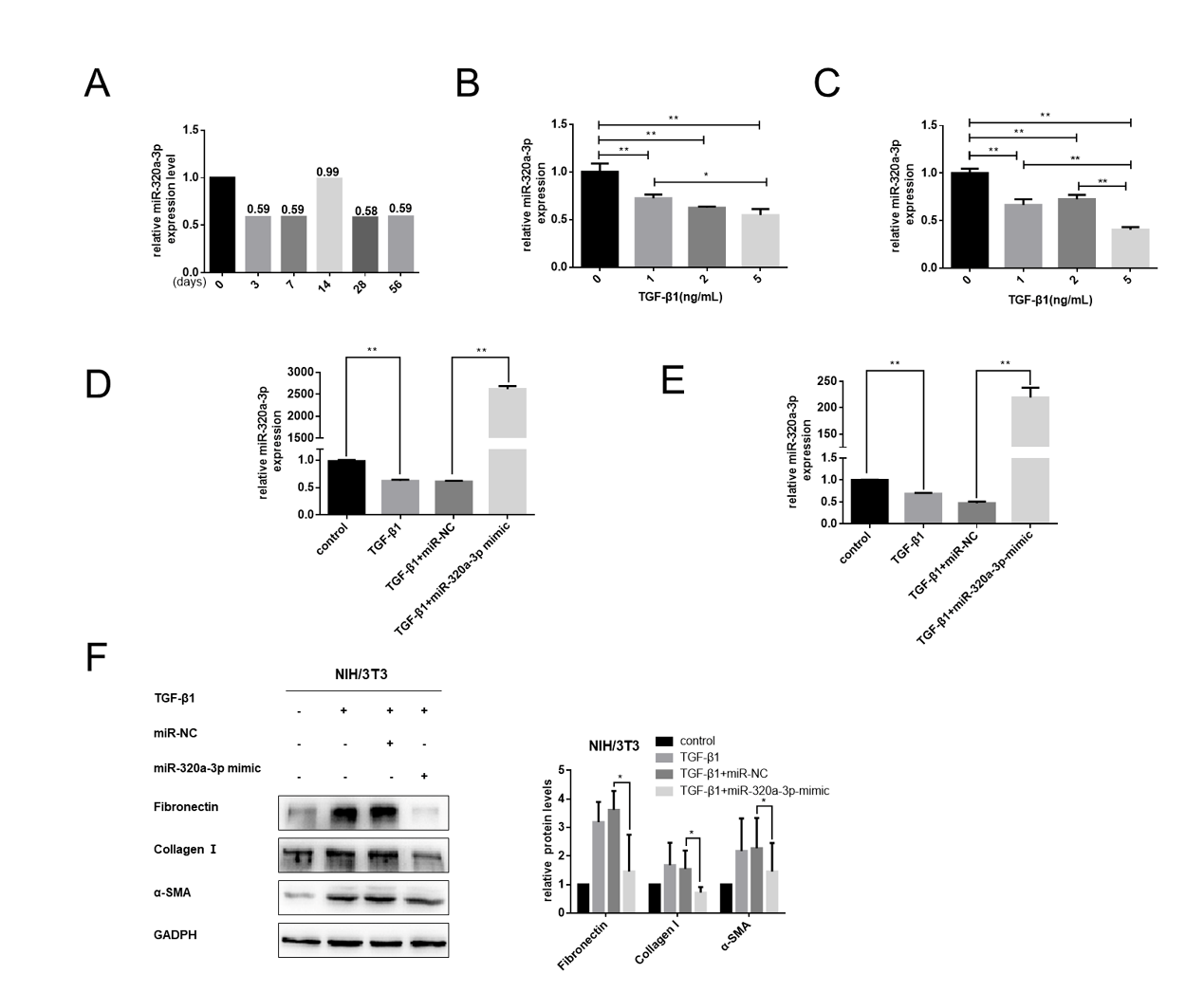


**Figure S3. miR-320a-3p is involved in the pathogenesis of silica-induced pulmonary fibrosis**

(A) The C57BL/6 mice were sacrificed on days 3, 7, 14, 28, 56 days after intratracheal instillation of silica suspended saline and saline. The relative expression of miR-320a-3p in mouse tissues was observed by microarray analysis. (B) and (C) miR-320a-3p was downregulated in TGF-β1-induced fibroblast activation, as evaluated by qRT-PCR. (D) and (E) qRT-PCR was used to measure levels of miR-320a-3p after treated with mimic and TGF-β1 in MRC-5 and NIH/3T3 cells. (F) Western blotting analysis the relative protein levels of MRC-5 cells transfected with 20 nM of miR-NC or Mir-320a-3p mimics before treated with 5 ng/mL of TGF-β1 for 48 h. All data were expressed as the means ± SD of at least 3 independent experiments, **p* < 0.05 and ***p* < 0.01.
