# Supplementary material for: ALKBH5 promotes lung fibroblast activation and silica-induced pulmonary fibrosis through miR-320a-3p and FOXM1
Source: Cell Mol Biol Lett. 2022 Mar 12;27:26. doi: 10.1186/s11658-022-00329-5 (PMC8917683; doi:10.1186/s11658-022-00329-5)
Supplement: Supplementary file 4 — Additional file 4: Fig. S4. FOXM1 is the direct and functional target of miR-320a-3p. [file 11658_2022_329_MOESM4_ESM.docx]

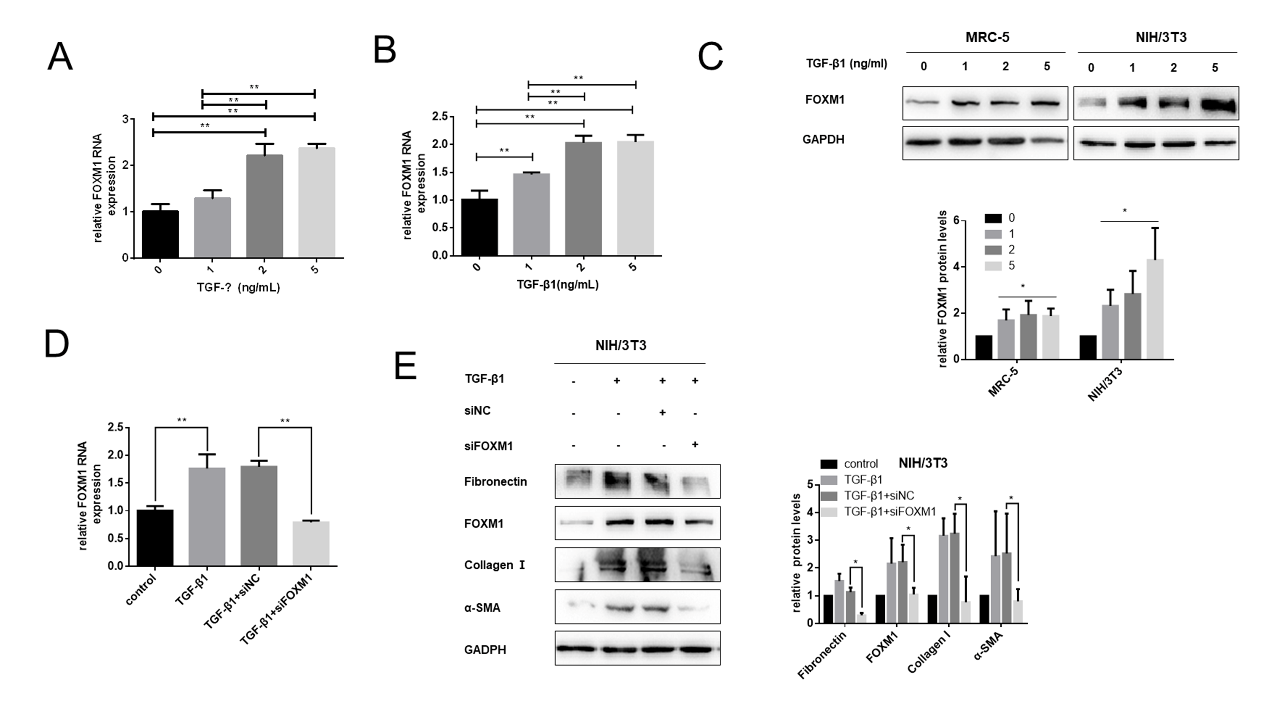


**Figure S4. FOXM1 is the direct and functional target of miR-320a-3p**

(A)-(C) FOXM1 was upregulated in TGF-β1-induced MRC-5 and NIH/3T3 cells in mRNA and protein levels, as measured by qRT-PCR and western blot analysis. (D) Transfection of siFOXM1 significantly decreased FOXM1 mRNA expression in NIH/3T3 cells treated with 5 ng/ml TGF-β1 for 48 h. (E) Western blotting analysis the relative protein levels of MRC-5 cells transfected with 50 nM of siNC or siFOXM1 before treated with 5 ng/mL of TGF-β1 for 48 h. All data were expressed as the means ± SD of at least 3 independent experiments, **p* < 0.05 and ***p* < 0.01.
