# Supplementary material for: ALKBH5 promotes lung fibroblast activation and silica-induced pulmonary fibrosis through miR-320a-3p and FOXM1
Source: Cell Mol Biol Lett. 2022 Mar 12;27:26. doi: 10.1186/s11658-022-00329-5 (PMC8917683; doi:10.1186/s11658-022-00329-5)
Supplement: Supplementary file 5 — Additional file 5: Fig. S5. Overexpression of miR-320a-3p exerts antifibrotic effects both in vitro and in vivo by targeting FOXM1. [file 11658_2022_329_MOESM5_ESM.docx]

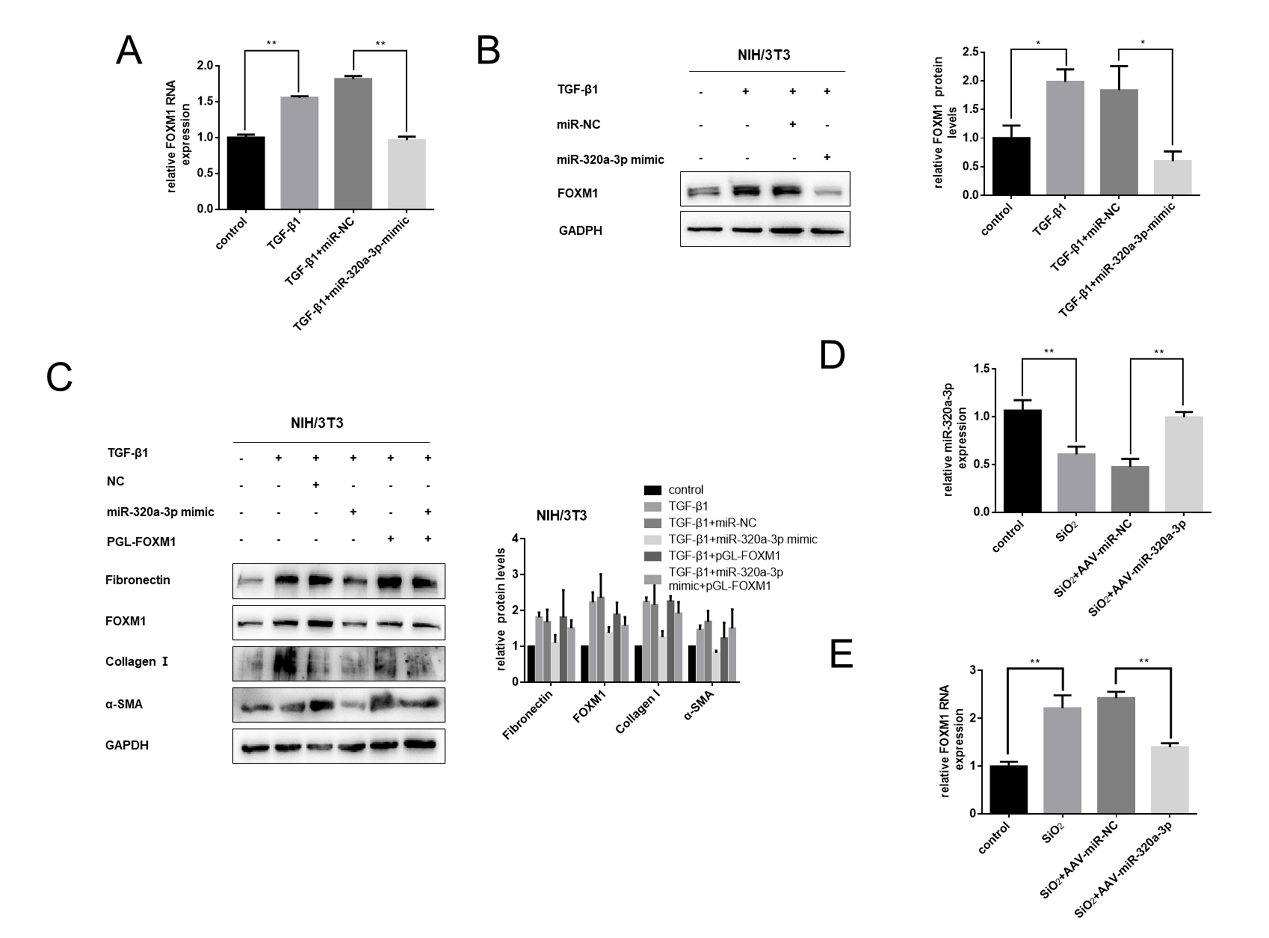


**Figure S5. Overexpression of miR-320a-3p exerts antifibrotic effects both in vitro and in vivo by targeting FOXM1**

(A) qRT-PCR and (B) western blot analysis showing that enhanced miR-320a-3p expression inhibited FOXM1 expression induced by TGF-β1 in NIH/3T3. (C) Western bolt showing that overexpression of FOXM1 significantly counteracted the inhibitory effects of miR-320a-3p in TGF-β1-induced fibroblast activation. All data were expressed as the means ± SD of at least 3 independent experiments, **p* < 0.05 and ***p* < 0.01.
