# Supplementary material for: ALKBH5 promotes lung fibroblast activation and silica-induced pulmonary fibrosis through miR-320a-3p and FOXM1
Source: Cell Mol Biol Lett. 2022 Mar 12;27:26. doi: 10.1186/s11658-022-00329-5 (PMC8917683; doi:10.1186/s11658-022-00329-5)
Supplement: Supplementary file 6 — Additional file 6: Table S1. Histologic scores for the severity and distribution of lung lesions. [file 11658_2022_329_MOESM6_ESM.docx]

**Table S1. Histologic scores about the severity and distribution of lung lesions**

| **Groups** | **Lesion severity**  **grade** | | | | | | | | **Average severity**  **grade** | **Lesion distribution**  **grade** | | | | | |  | **Average**  **distribution**  **grade** |  |
| --- | --- | --- | --- | --- | --- | --- | --- | --- | --- | --- | --- | --- | --- | --- | --- | --- | --- | --- |
|  | 0 | 1 | 2 | 3 | 4 | 5 |  | | | 0 | 1 | 2 | 3 | 4 | 5 |  |  | |
| **Saline** | 6 |  |  |  |  |  | |  | | 6 |  |  |  |  |  |  | 0 | |
| **silica** |  |  |  |  | 3 | 3 | | 4.50±0.548 | |  |  |  | 4 | 1 | 1 |  | 3.50±0.837 | |
| **Silica + AAV-miR-NC** |  |  |  |  | 2 | 4 | | 4.67±0.516 | |  |  |  | 2 | 3 | 1 |  | 3.83±0.753 | |
| **Silica+AAV-miR-320a-3p** | 2 | 1 | 3 |  |  |  | | 2.17±0.983* | | 2 |  | 2 | 2 |  |  |  | 2.00±0.894* | |

Note: Values represent the means ± SD; **p* < 0.05 vs. silica + AAV-miR-NC group (n = 6 in each group, independent samples *t-*test).
