# Supplementary material for: ALKBH5 promotes lung fibroblast activation and silica-induced pulmonary fibrosis through miR-320a-3p and FOXM1
Source: Cell Mol Biol Lett. 2022 Mar 12;27:26. doi: 10.1186/s11658-022-00329-5 (PMC8917683; doi:10.1186/s11658-022-00329-5)
Supplement: Supplementary file 7 — Additional file 7. Original images for western blots. [file 11658_2022_329_MOESM7_ESM.pptx]

## Slide 1
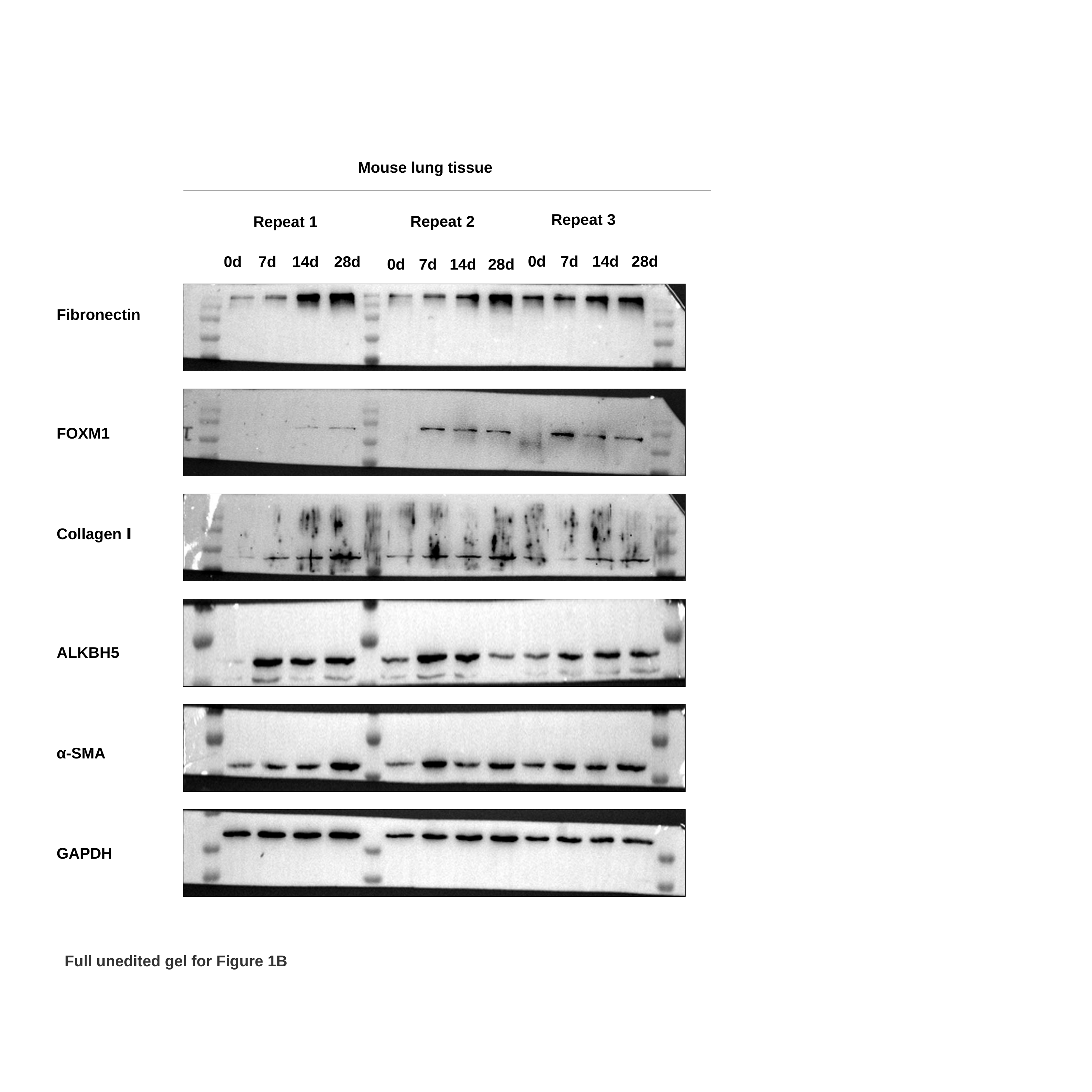

Mouse lung tissue
Repeat 3
Repeat 2
Repeat 1
| 0d | 7d | 14d | 28d |
| --- | --- | --- | --- |
| 0d | 7d | 14d | 28d |
| --- | --- | --- | --- |
| 0d | 7d | 14d | 28d |
| --- | --- | --- | --- |
Fibronectin
FOXM1
Collagen Ⅰ
ALKBH5
α-SMA
GAPDH
Full unedited gel for Figure 1B

## Slide 2
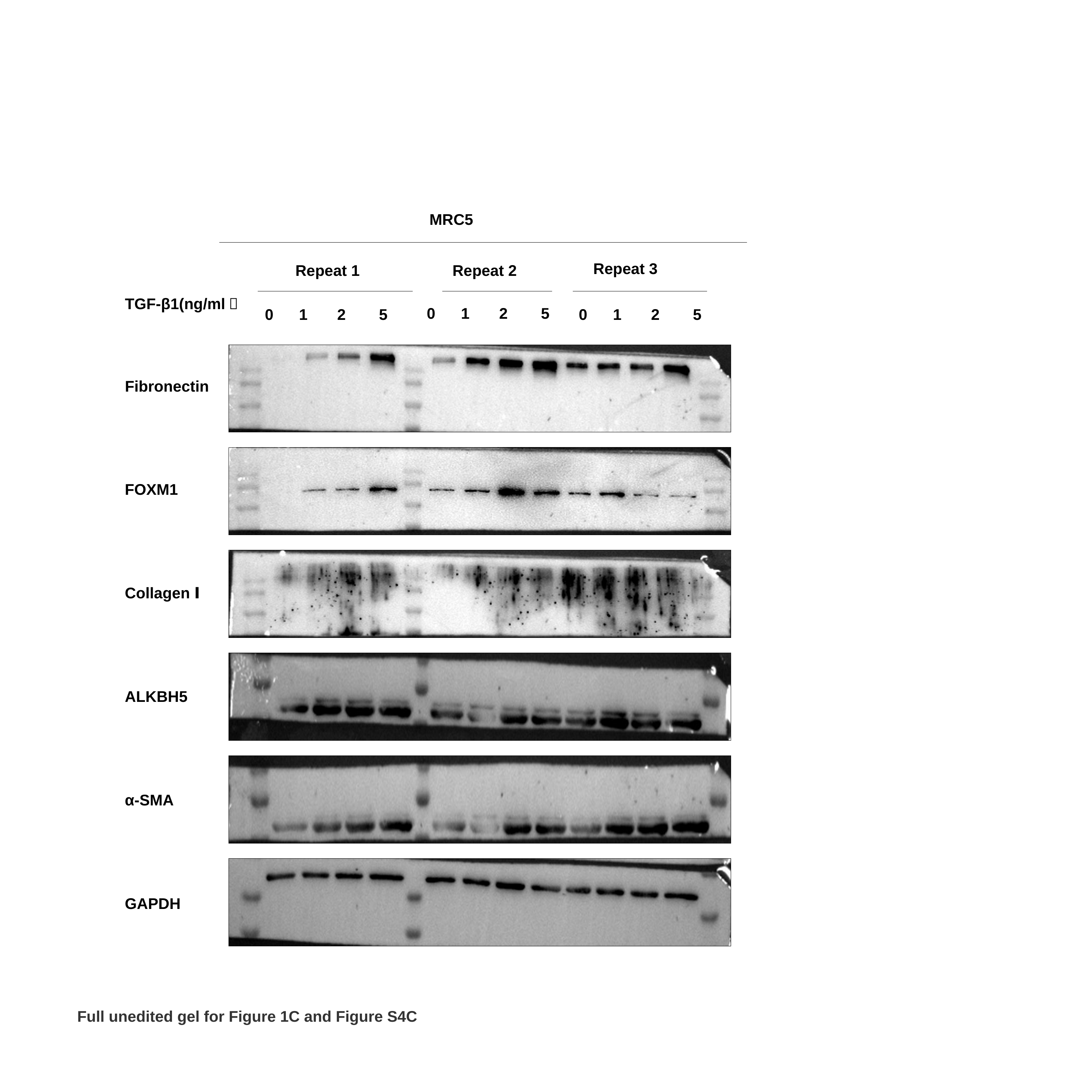

MRC5
Repeat 3
Repeat 2
Repeat 1
TGF-β1(ng/ml）
| 0 | 1 | 2 | 5 |
| --- | --- | --- | --- |
| 0 | 1 | 2 | 5 |
| --- | --- | --- | --- |
| 0 | 1 | 2 | 5 |
| --- | --- | --- | --- |
Fibronectin
FOXM1
Collagen Ⅰ
ALKBH5
α-SMA
GAPDH
Full unedited gel for Figure 1C and Figure S4C

## Slide 3
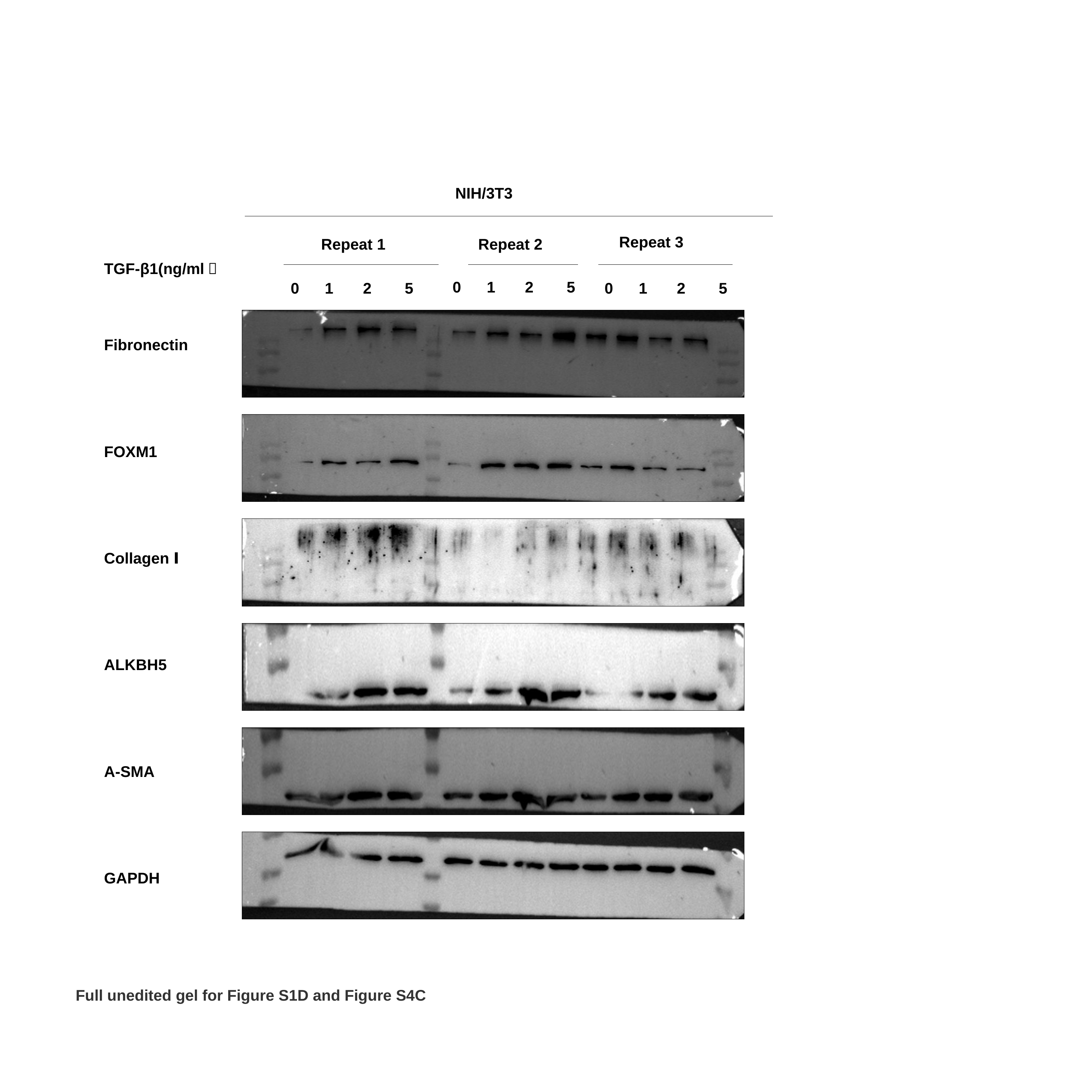

NIH/3T3
Repeat 3
Repeat 2
Repeat 1
TGF-β1(ng/ml）
| 0 | 1 | 2 | 5 |
| --- | --- | --- | --- |
| 0 | 1 | 2 | 5 |
| --- | --- | --- | --- |
| 0 | 1 | 2 | 5 |
| --- | --- | --- | --- |
Fibronectin
FOXM1
Collagen Ⅰ
ALKBH5
Α-SMA
GAPDH
Full unedited gel for Figure S1D and Figure S4C

## Slide 4
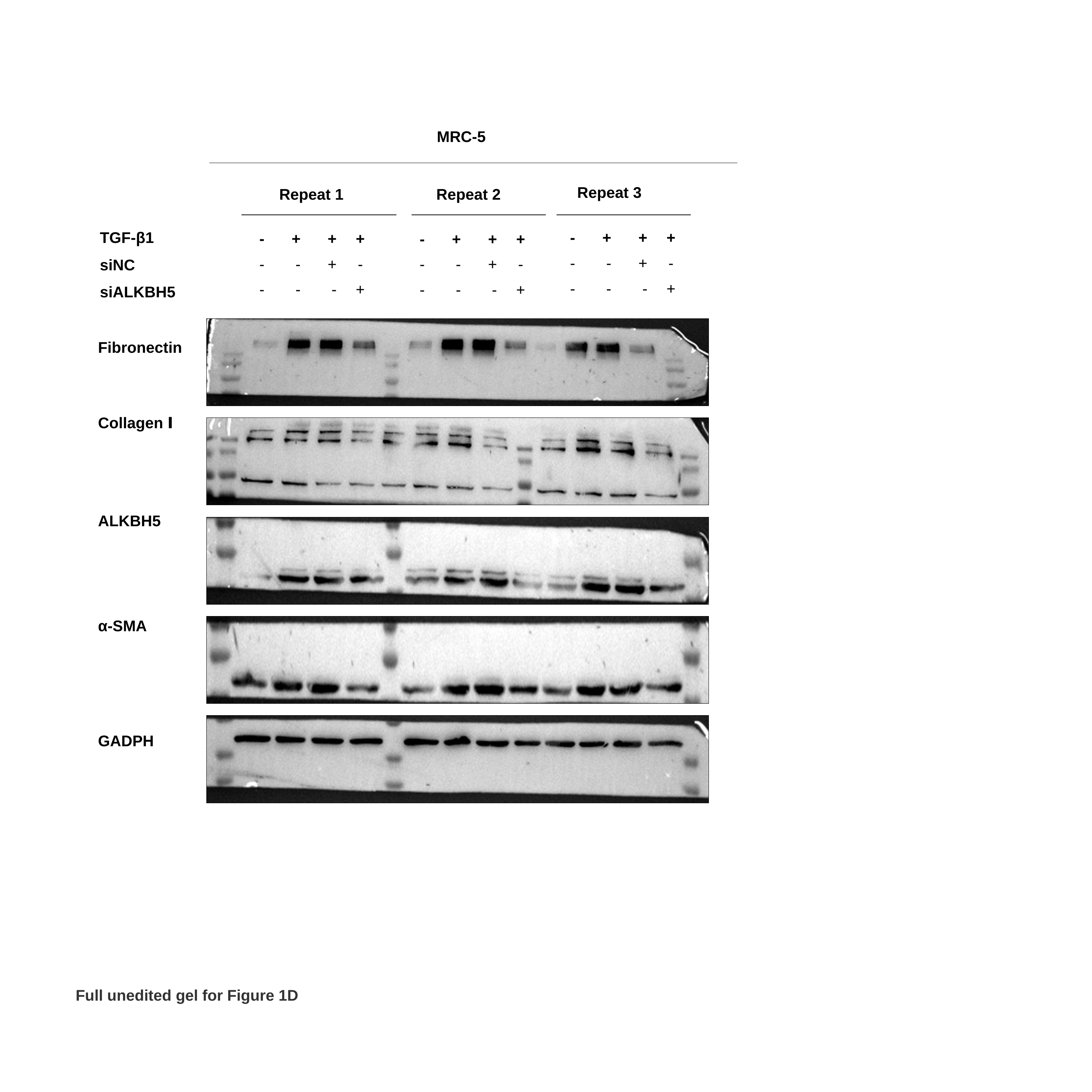

MRC-5
Repeat 3
Repeat 2
Repeat 1
TGF-β1
| - | + | + | + |
| --- | --- | --- | --- |
| - | - | + | - |
| - | - | - | + |
| - | + | + | + |
| --- | --- | --- | --- |
| - | - | + | - |
| - | - | - | + |
| - | + | + | + |
| --- | --- | --- | --- |
| - | - | + | - |
| - | - | - | + |
siNC
siALKBH5
Fibronectin
Collagen Ⅰ
ALKBH5
α-SMA
GADPH
Full unedited gel for Figure 1D

## Slide 5
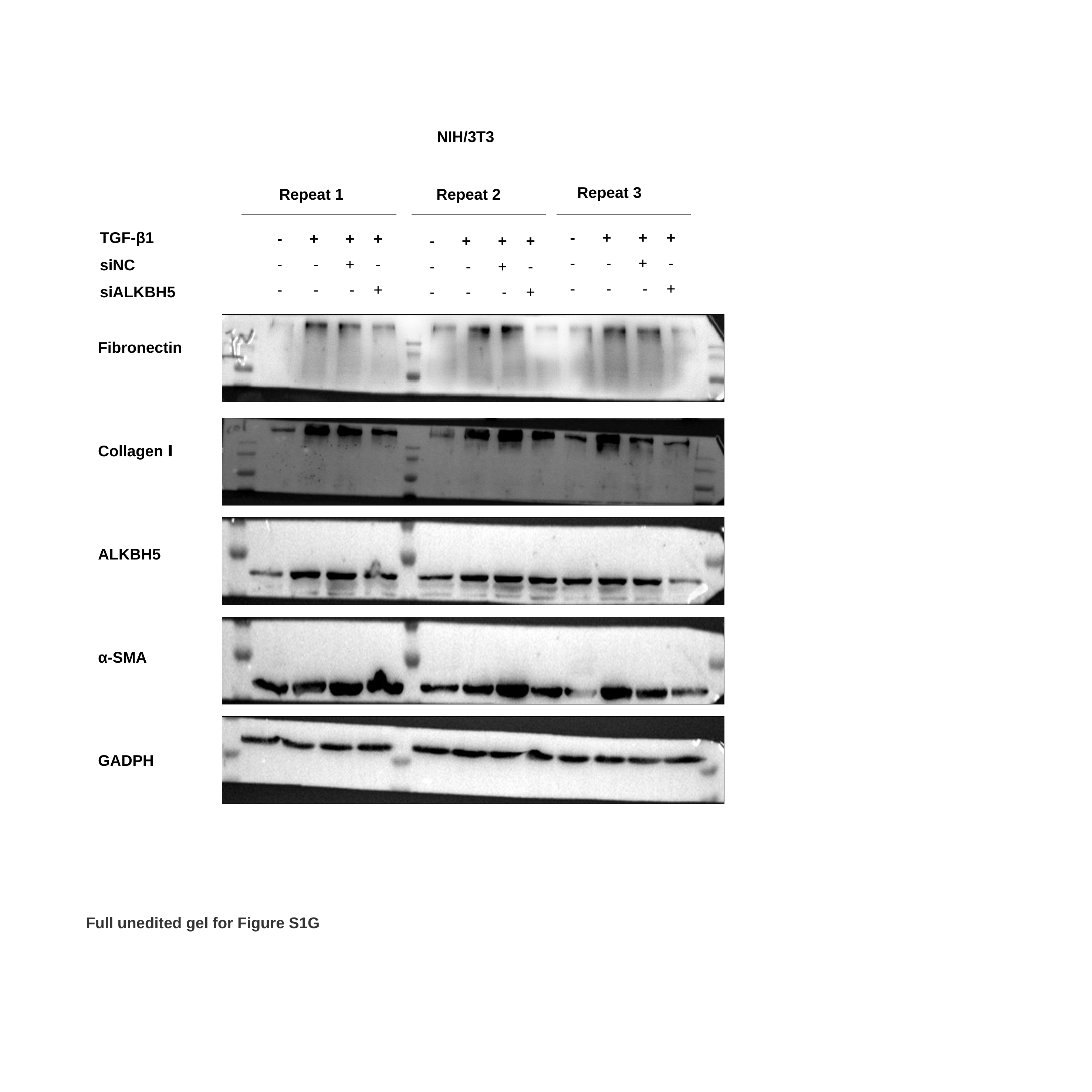

NIH/3T3
Repeat 3
Repeat 2
Repeat 1
TGF-β1
| - | + | + | + |
| --- | --- | --- | --- |
| - | - | + | - |
| - | - | - | + |
| - | + | + | + |
| --- | --- | --- | --- |
| - | - | + | - |
| - | - | - | + |
| - | + | + | + |
| --- | --- | --- | --- |
| - | - | + | - |
| - | - | - | + |
siNC
siALKBH5
Fibronectin
Collagen Ⅰ
ALKBH5
α-SMA
GADPH
Full unedited gel for Figure S1G

## Slide 6
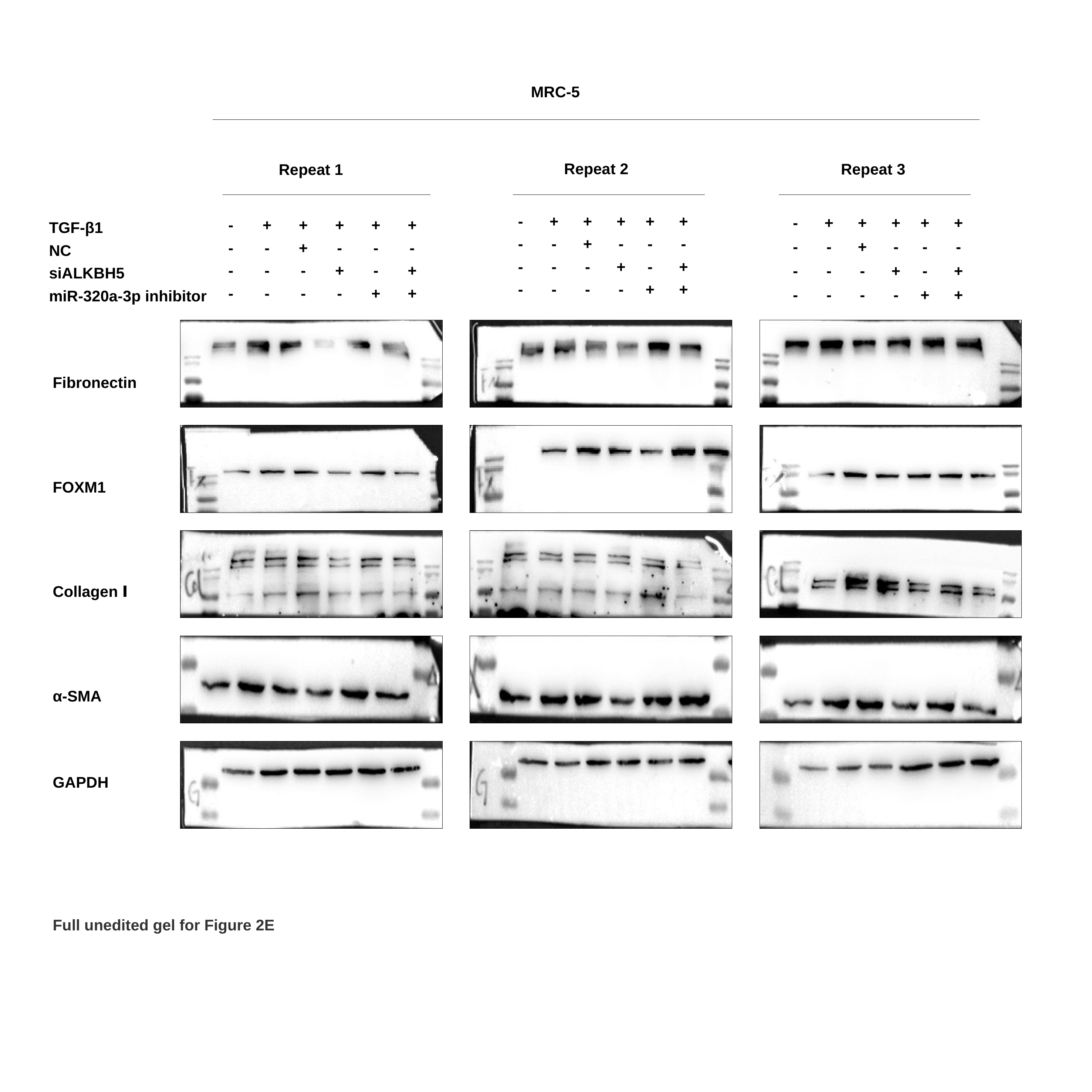

MRC-5
Repeat 2
Repeat 3
Repeat 1
| - | + | + | + | + | + |
| --- | --- | --- | --- | --- | --- |
| - | - | + | - | - | - |
| - | - | - | + | - | + |
| - | - | - | - | + | + |
| - | + | + | + | + | + |
| --- | --- | --- | --- | --- | --- |
| - | - | + | - | - | - |
| - | - | - | + | - | + |
| - | - | - | - | + | + |
| - | + | + | + | + | + |
| --- | --- | --- | --- | --- | --- |
| - | - | + | - | - | - |
| - | - | - | + | - | + |
| - | - | - | - | + | + |
TGF-β1
NC
siALKBH5
miR-320a-3p inhibitor
Fibronectin
FOXM1
Collagen Ⅰ
α-SMA
GAPDH
Full unedited gel for Figure 2E

## Slide 7
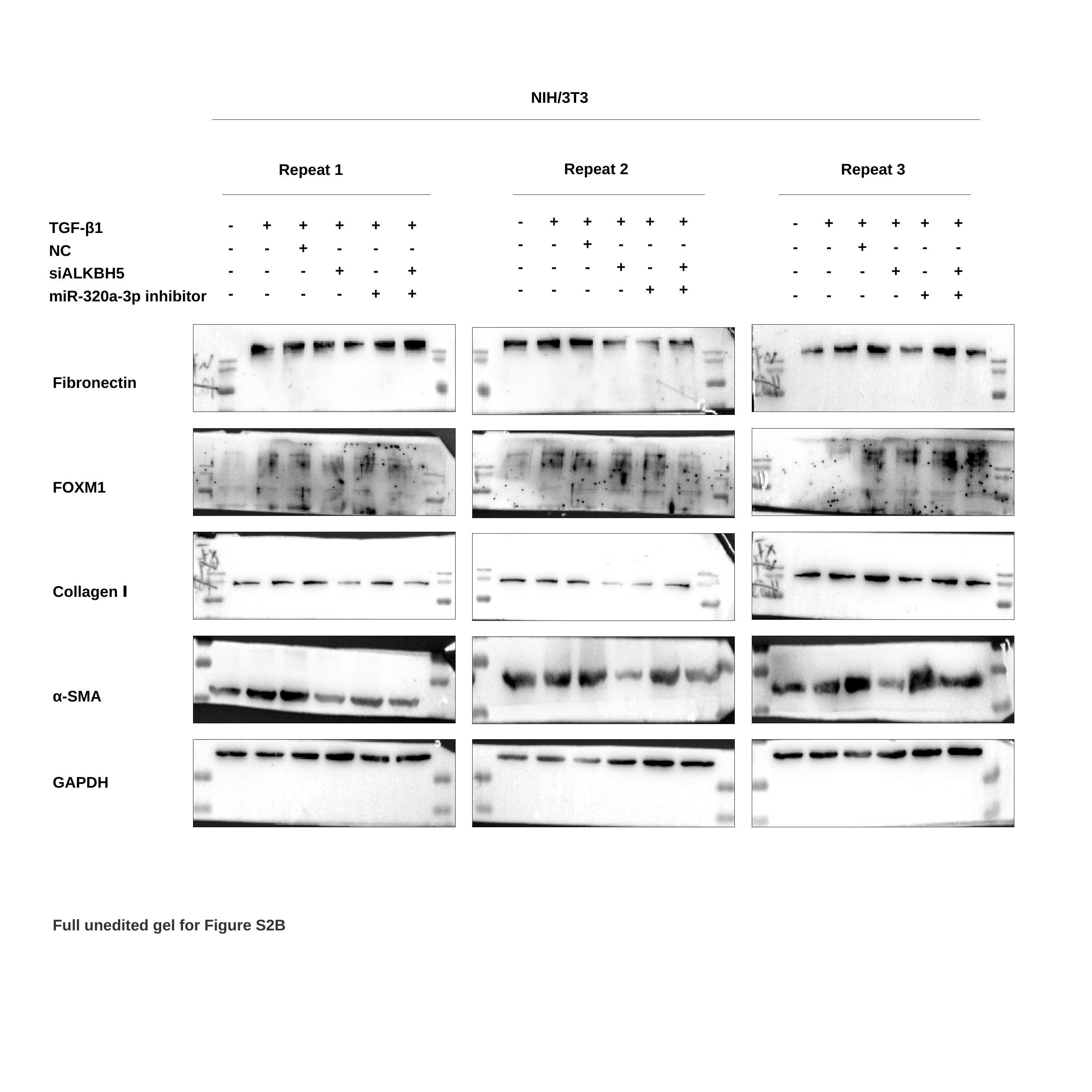

NIH/3T3
Repeat 2
Repeat 3
Repeat 1
| - | + | + | + | + | + |
| --- | --- | --- | --- | --- | --- |
| - | - | + | - | - | - |
| - | - | - | + | - | + |
| - | - | - | - | + | + |
| - | + | + | + | + | + |
| --- | --- | --- | --- | --- | --- |
| - | - | + | - | - | - |
| - | - | - | + | - | + |
| - | - | - | - | + | + |
| - | + | + | + | + | + |
| --- | --- | --- | --- | --- | --- |
| - | - | + | - | - | - |
| - | - | - | + | - | + |
| - | - | - | - | + | + |
TGF-β1
NC
siALKBH5
miR-320a-3p inhibitor
Fibronectin
FOXM1
Collagen Ⅰ
α-SMA
GAPDH
Full unedited gel for Figure S2B

## Slide 8
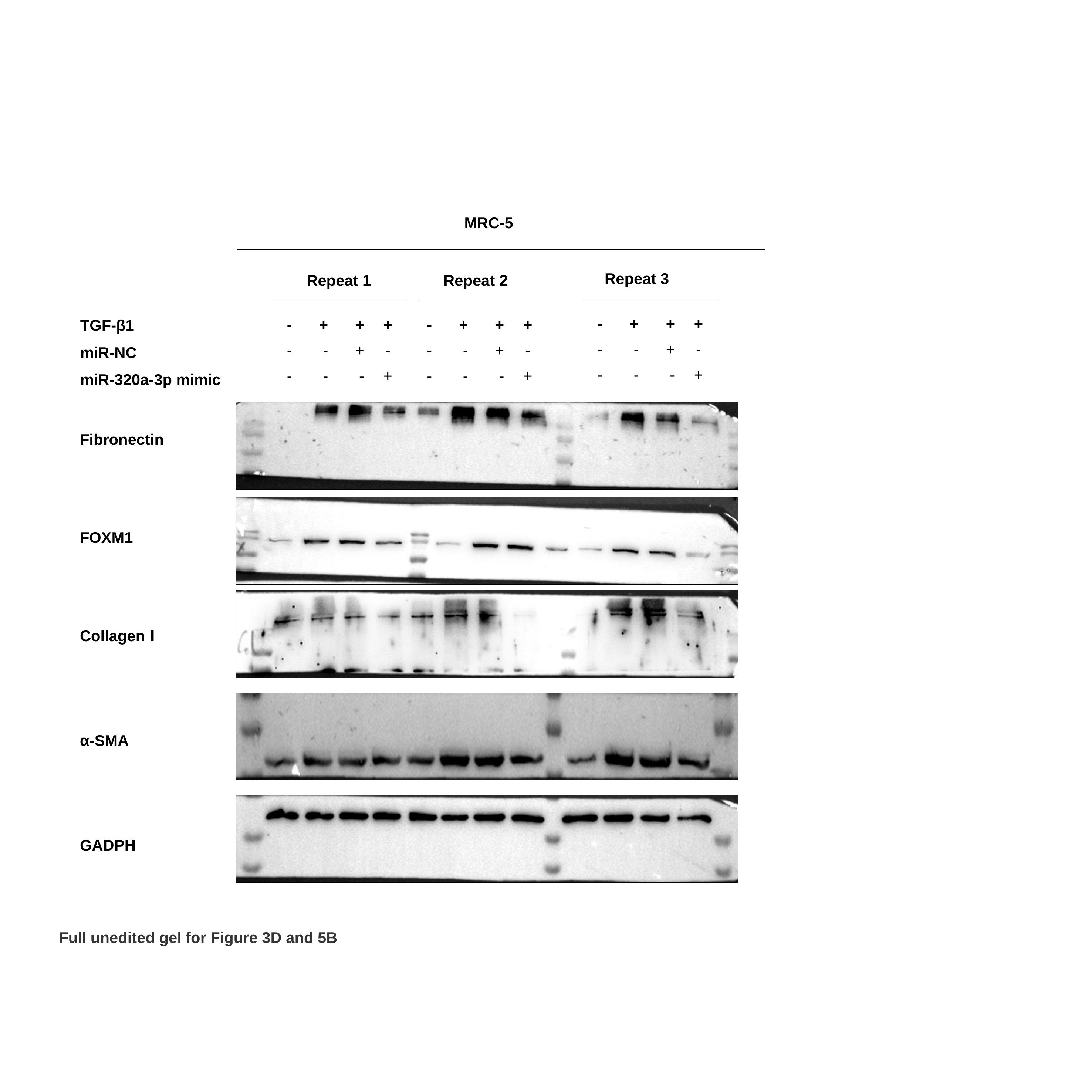

MRC-5
Repeat 3
Repeat 2
Repeat 1
TGF-β1
| - | + | + | + |
| --- | --- | --- | --- |
| - | - | + | - |
| - | - | - | + |
| - | + | + | + |
| --- | --- | --- | --- |
| - | - | + | - |
| - | - | - | + |
| - | + | + | + |
| --- | --- | --- | --- |
| - | - | + | - |
| - | - | - | + |
miR-NC
miR-320a-3p mimic
Fibronectin
FOXM1
Collagen Ⅰ
α-SMA
GADPH
Full unedited gel for Figure 3D and 5B

## Slide 9
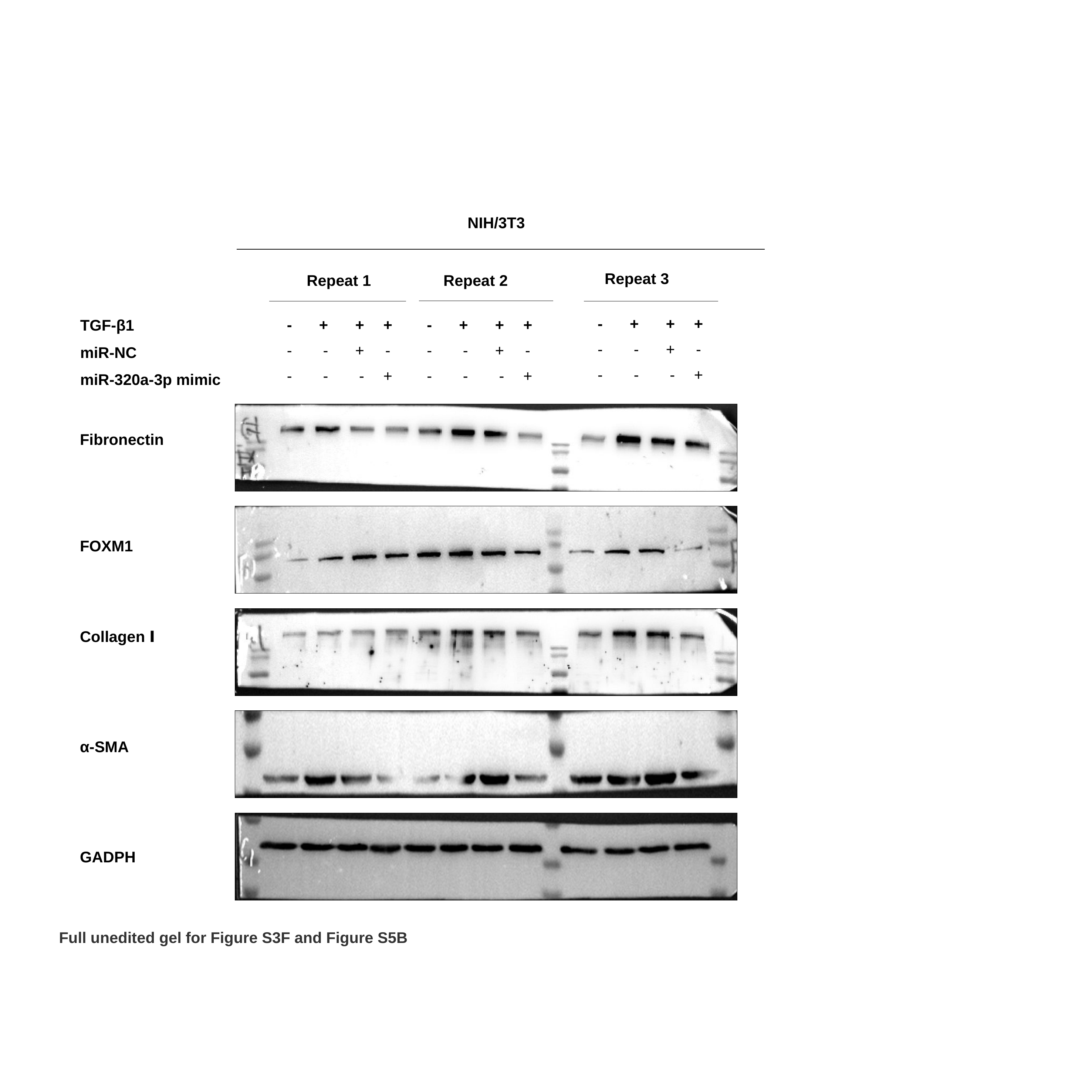

NIH/3T3
Repeat 3
Repeat 2
Repeat 1
TGF-β1
| - | + | + | + |
| --- | --- | --- | --- |
| - | - | + | - |
| - | - | - | + |
| - | + | + | + |
| --- | --- | --- | --- |
| - | - | + | - |
| - | - | - | + |
| - | + | + | + |
| --- | --- | --- | --- |
| - | - | + | - |
| - | - | - | + |
miR-NC
miR-320a-3p mimic
Fibronectin
FOXM1
Collagen Ⅰ
α-SMA
GADPH
Full unedited gel for Figure S3F and Figure S5B

## Slide 10
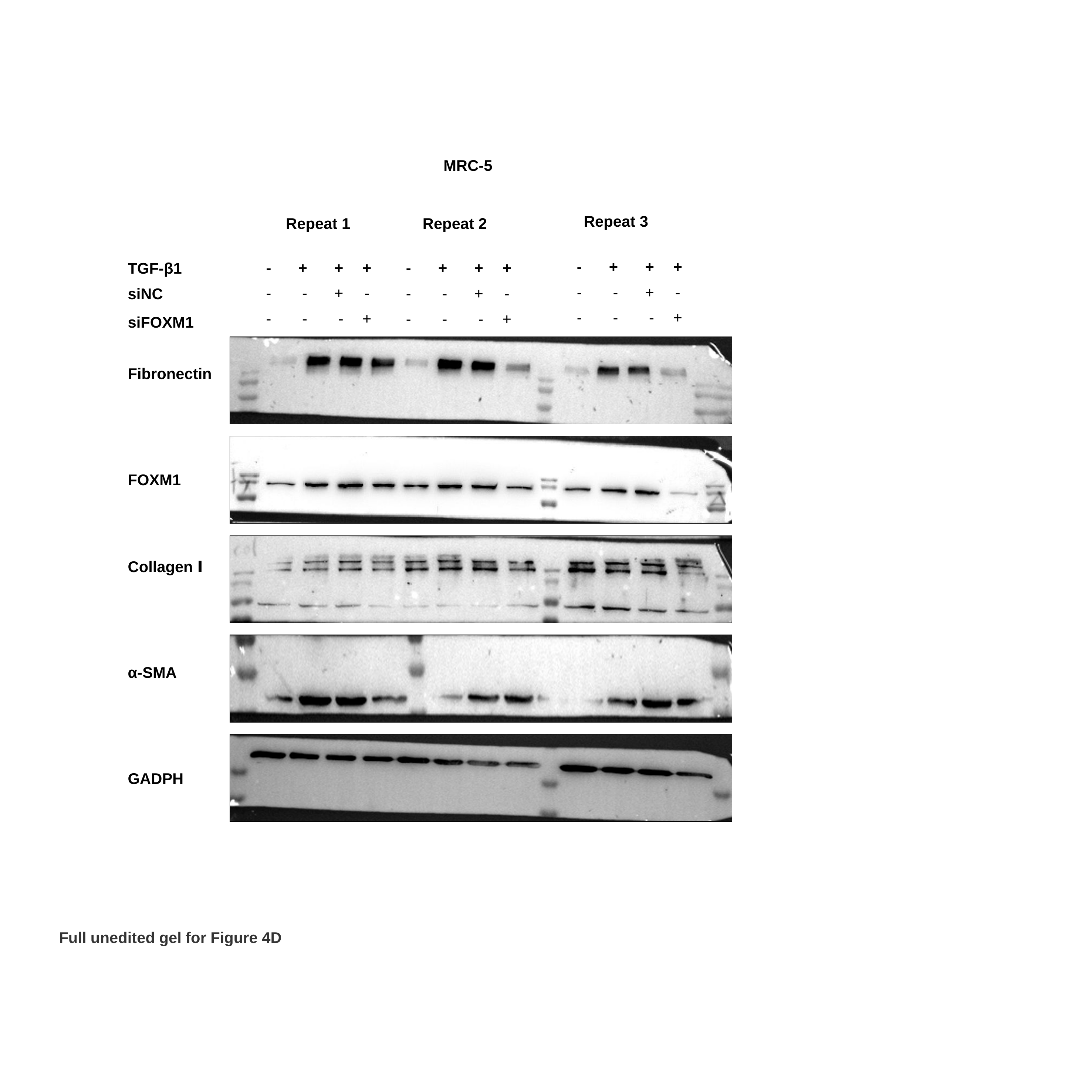

MRC-5
Repeat 3
Repeat 2
Repeat 1
TGF-β1
| - | + | + | + |
| --- | --- | --- | --- |
| - | - | + | - |
| - | - | - | + |
| - | + | + | + |
| --- | --- | --- | --- |
| - | - | + | - |
| - | - | - | + |
| - | + | + | + |
| --- | --- | --- | --- |
| - | - | + | - |
| - | - | - | + |
siNC
siFOXM1
Fibronectin
FOXM1
Collagen Ⅰ
α-SMA
GADPH
Full unedited gel for Figure 4D

## Slide 11
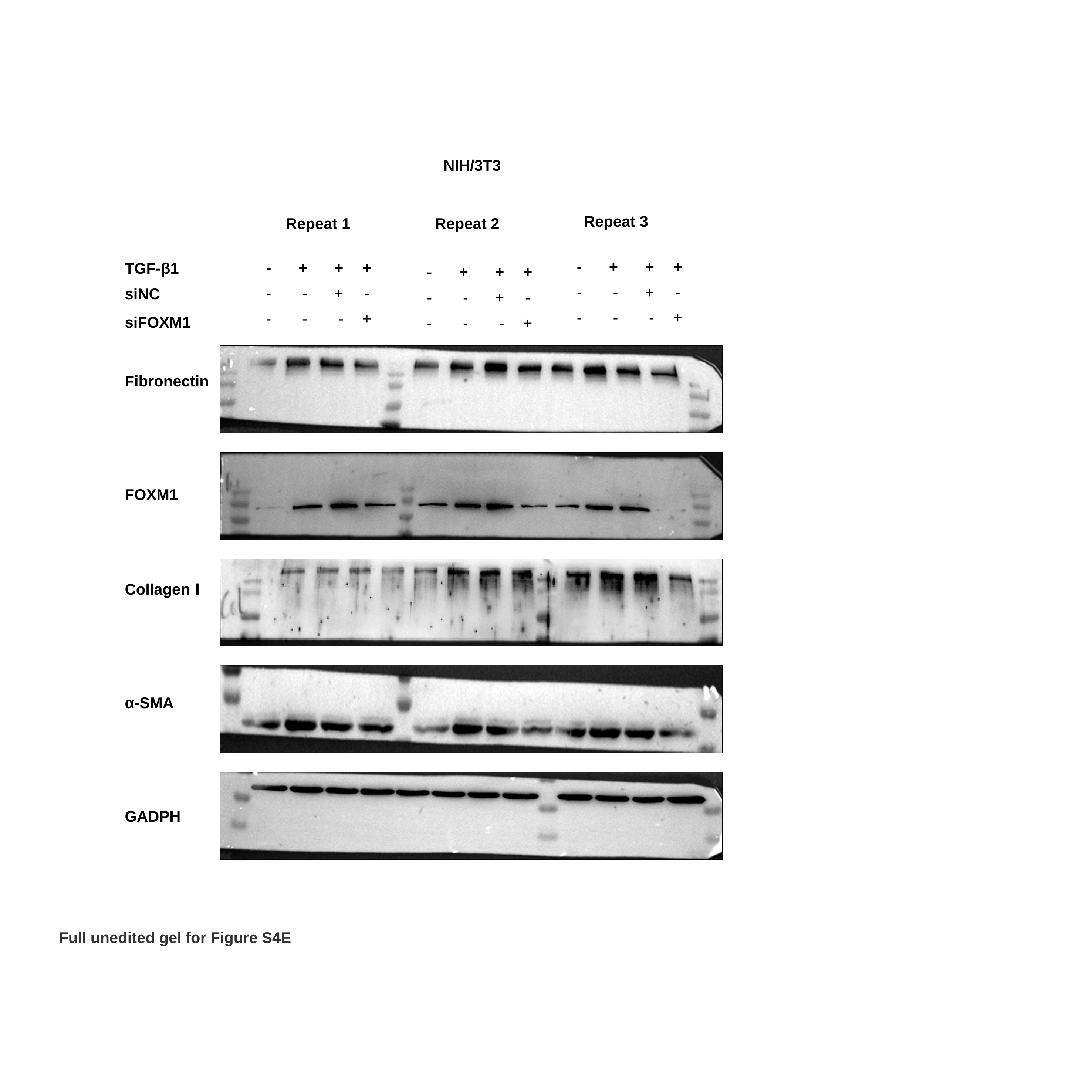

NIH/3T3
Repeat 3
Repeat 1
Repeat 2
TGF-β1
| - | + | + | + |
| --- | --- | --- | --- |
| - | - | + | - |
| - | - | - | + |
| - | + | + | + |
| --- | --- | --- | --- |
| - | - | + | - |
| - | - | - | + |
| - | + | + | + |
| --- | --- | --- | --- |
| - | - | + | - |
| - | - | - | + |
siNC
siFOXM1
Fibronectin
FOXM1
Collagen Ⅰ
α-SMA
GADPH
Full unedited gel for Figure S4E

## Slide 12
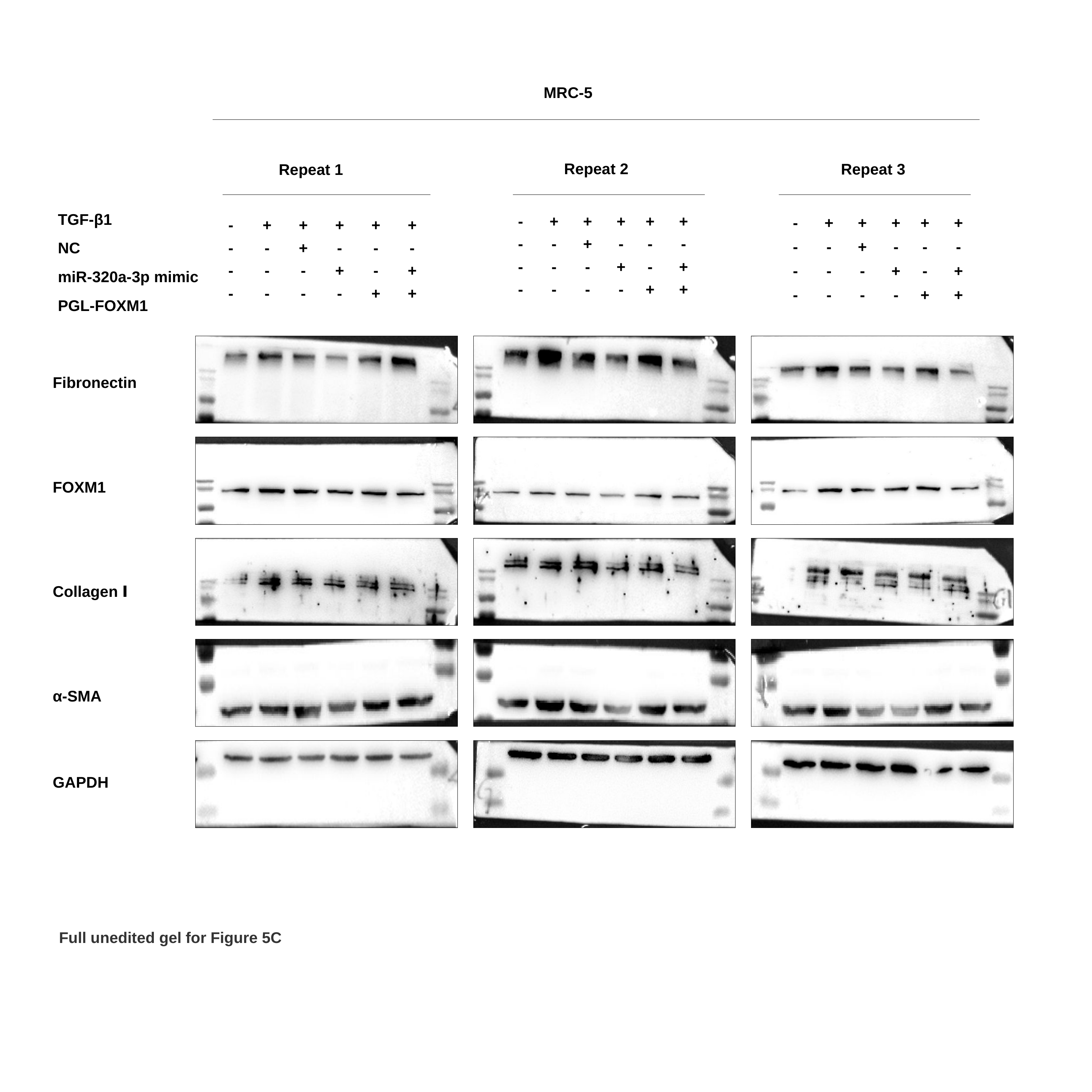

MRC-5
Repeat 2
Repeat 3
Repeat 1
TGF-β1
| - | + | + | + | + | + |
| --- | --- | --- | --- | --- | --- |
| - | - | + | - | - | - |
| - | - | - | + | - | + |
| - | - | - | - | + | + |
| - | + | + | + | + | + |
| --- | --- | --- | --- | --- | --- |
| - | - | + | - | - | - |
| - | - | - | + | - | + |
| - | - | - | - | + | + |
| - | + | + | + | + | + |
| --- | --- | --- | --- | --- | --- |
| - | - | + | - | - | - |
| - | - | - | + | - | + |
| - | - | - | - | + | + |
NC
miR-320a-3p mimic
PGL-FOXM1
Fibronectin
FOXM1
Collagen Ⅰ
α-SMA
GAPDH
Full unedited gel for Figure 5C

## Slide 13
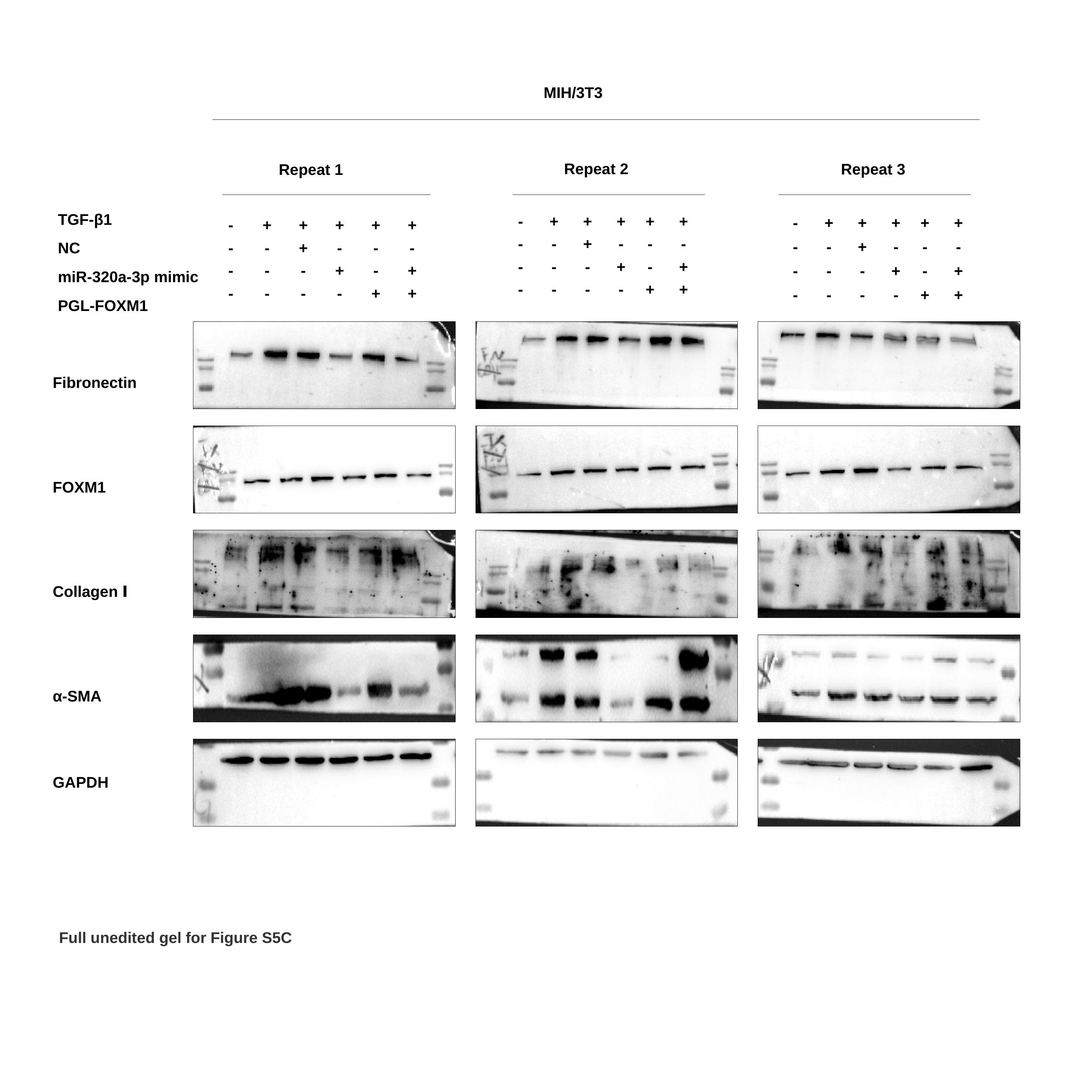

MIH/3T3
Repeat 2
Repeat 3
Repeat 1
TGF-β1
| - | + | + | + | + | + |
| --- | --- | --- | --- | --- | --- |
| - | - | + | - | - | - |
| - | - | - | + | - | + |
| - | - | - | - | + | + |
| - | + | + | + | + | + |
| --- | --- | --- | --- | --- | --- |
| - | - | + | - | - | - |
| - | - | - | + | - | + |
| - | - | - | - | + | + |
| - | + | + | + | + | + |
| --- | --- | --- | --- | --- | --- |
| - | - | + | - | - | - |
| - | - | - | + | - | + |
| - | - | - | - | + | + |
NC
miR-320a-3p mimic
PGL-FOXM1
Fibronectin
FOXM1
Collagen Ⅰ
α-SMA
GAPDH
Full unedited gel for Figure S5C

## Slide 14
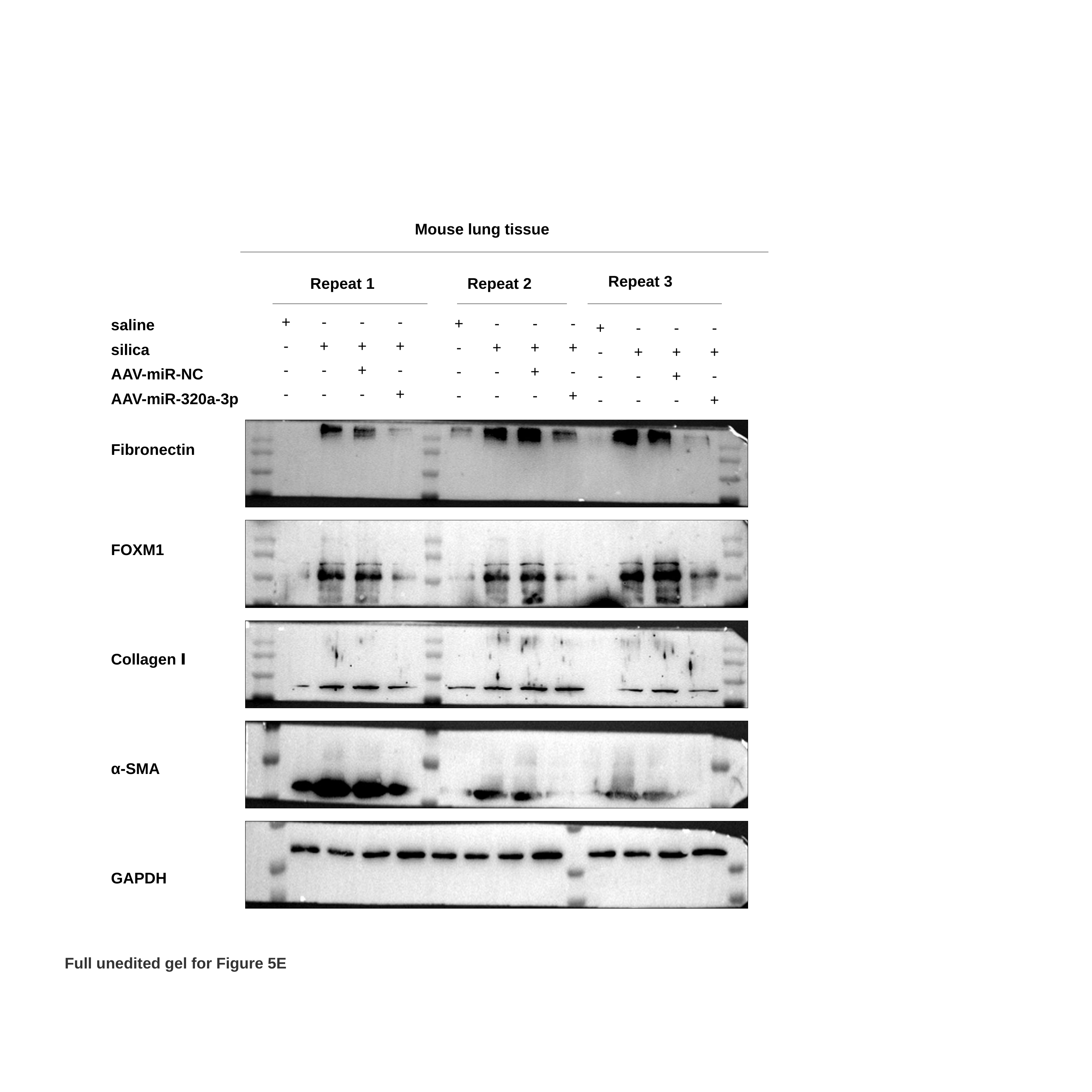

Mouse lung tissue
Repeat 3
Repeat 2
Repeat 1
| + | - | - | - |
| --- | --- | --- | --- |
| - | + | + | + |
| - | - | + | - |
| - | - | - | + |
saline
| + | - | - | - |
| --- | --- | --- | --- |
| - | + | + | + |
| - | - | + | - |
| - | - | - | + |
| + | - | - | - |
| --- | --- | --- | --- |
| - | + | + | + |
| - | - | + | - |
| - | - | - | + |
silica
AAV-miR-NC
AAV-miR-320a-3p
Fibronectin
FOXM1
Collagen Ⅰ
α-SMA
GAPDH
Full unedited gel for Figure 5E

## Slide 15
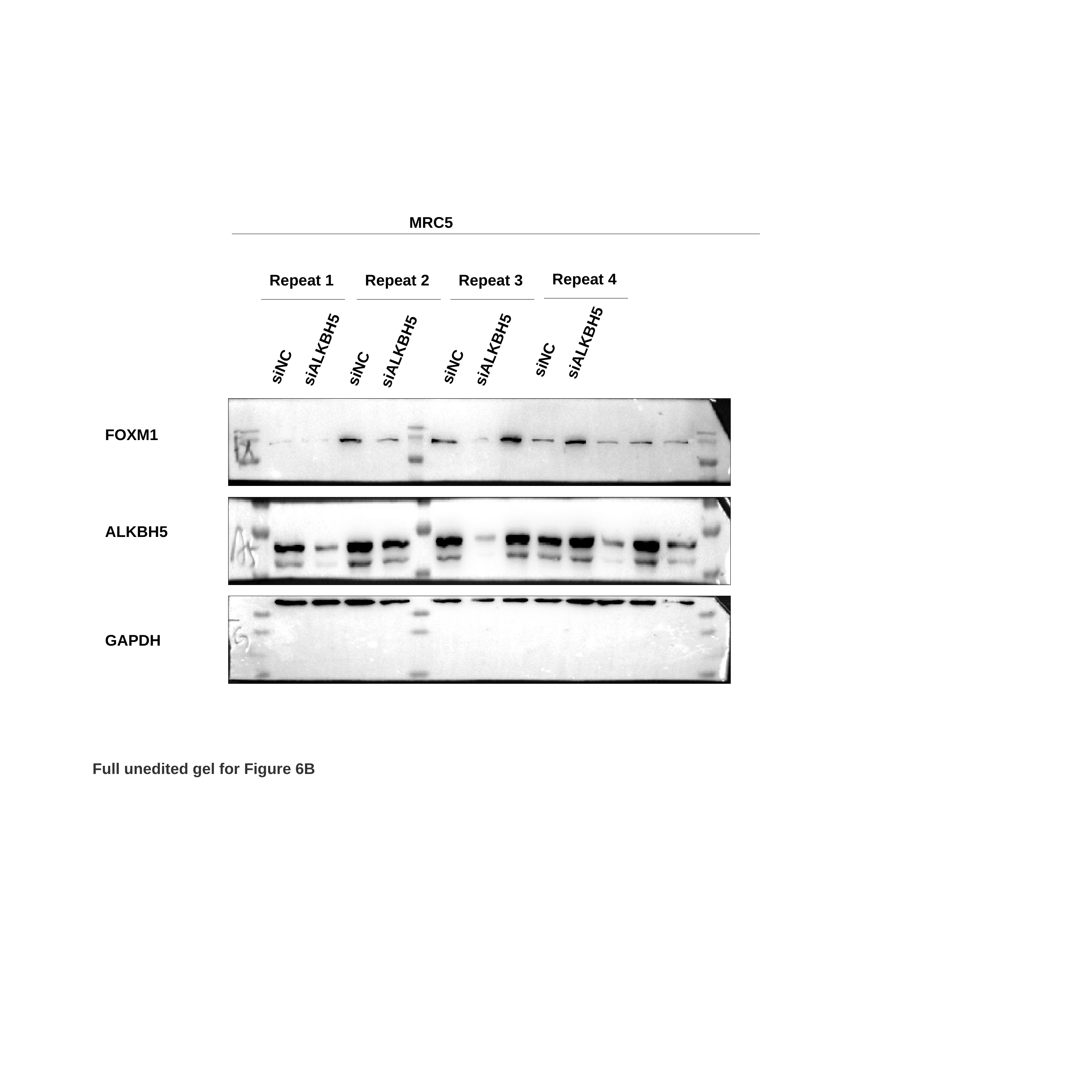

MRC5
Repeat 4
Repeat 1
Repeat 2
Repeat 3
siALKBH5
siALKBH5
siALKBH5
siALKBH5
siNC
siNC
siNC
siNC
FOXM1
ALKBH5
GAPDH
Full unedited gel for Figure 6B
